# Complementary water and nutrient utilization of perianth structural units help maintain long floral lifespan in *Dendrobium*

**DOI:** 10.1093/jxb/erac479

**Published:** 2022-12-03

**Authors:** Jia-Wei Li, Yi Zhou, Zi-Bin Zhang, Xue-Qiang Cui, Hong-Yan Li, Mei-Jing Ou, Kun-Fang Cao, Shi-Bao Zhang

**Affiliations:** State Key Laboratory for Conservation and Utilization of Subtropical Agro-bioresources, College of Forestry, Guangxi University, Nanning, Guangxi, 530004, China; State Key Laboratory for Conservation and Utilization of Subtropical Agro-bioresources, College of Forestry, Guangxi University, Nanning, Guangxi, 530004, China; Flower Research Institute, Guangxi Academy of Agricultural Sciences, Nanning, Guangxi 530007, China; Flower Research Institute, Guangxi Academy of Agricultural Sciences, Nanning, Guangxi 530007, China; State Key Laboratory for Conservation and Utilization of Subtropical Agro-bioresources, College of Forestry, Guangxi University, Nanning, Guangxi, 530004, China; State Key Laboratory for Conservation and Utilization of Subtropical Agro-bioresources, College of Forestry, Guangxi University, Nanning, Guangxi, 530004, China; State Key Laboratory for Conservation and Utilization of Subtropical Agro-bioresources, College of Forestry, Guangxi University, Nanning, Guangxi, 530004, China; Key Laboratory for Economic Plants and Biotechnology, Kunming Institute of Botany, Chinese Academy of Sciences, Kunming, Yunnan 650201, China; Swedish University of Agricultural Sciences, Sweden

**Keywords:** Biomechanical strength, *Dendrobium*, floral longevity, labellum, nutrient resorption, polysaccharide

## Abstract

Most orchids have high ornamental value with long-lived flowers. However, the mechanisms by which orchids maintain floral longevity are poorly understood. Here, we hypothesized that floral longevity in *Dendrobium* is maintained by high resource investment and complementary water and nutrient utilization in different structural units of the perianth. To test this hypothesis, we determined which water- and nutrient-related traits are correlated with flower longevity in 23 *Dendrobium* species or cultivars, and examined variations of the related traits during flower development of one long-lived cultivar. We found that floral longevity was correlated with dry mass per unit area of perianths and total flower biomass, which indicates that maintaining floral longevity requires increased resource investment. During development of long-lived flowers, labella showed a high capacity for water storage and nutrient reutilization, which could partly remedy high water demand and biomass investment. Sepals and petals, in contrast, had stronger desiccation avoidance and higher metabolic activity with lower biomass investment. These findings indicate that *Dendrobium* flowers maintain longevity by complementary water and nutrient utilization strategies in the sepals, petals and labella, with labella consuming more water and nutrients to extend flower display, and sepals and petals using a more conservative strategy.

## Teaser

The labella of *Dendrobium*flowers have higher nutrient requirements and metabolic activity compared with the sepals and petals, with higher capacity for water supply and nutrient reuse, thereby maintaining long floral lifespan.

## Introduction

The flowers of orchids have long fascinated naturalists, ecologists, and horticulturalists (He *et al.*, 2011; Hossain *et al.*, 2013). Orchid flower displays are critical for attracting specific pollinators, which are often rare in the habitats of most orchids. One strategy that orchids use to increase reproductive success is the prolongation of floral displays (Ashman *et al.*, 1994; van Doorn, 1997; Galen, 1999; Abdala-Roberts *et al.*, 2007; Vega and Marques, 2015). However, prolonged floral longevity is costly (Ashman *et al.*, 1994; Galen, 1999), especially in environments characterized by limited supplies of water and nutrients, like the epiphytic environment inhabited by 70% orchid species (Zotz and Hietz 2001; Laube and Zotz 2003; Arroyo *et al.*, 2013; Jorgensen and Arathi, 2013; Song *et al.*, 2022). Understanding the anatomical and physiological mechanisms that underlie the maintenance of long-lived flowers may provide insights into the ecological adaptation of orchids, and the production of new cultivars with long lifespan of flowers.

One major mechanism for prolonging flower display is maintaining water balance. For example, flower display has been shown to be significantly influenced by changes in cell turgor pressure (Teixido and Valladares, 2014; Roddy, 2019; Teixido *et al.*, 2019; Kuppler and Kotowska, 2021). The anatomical and physiological traits of flowers (e.g. low vein density, high hydraulic capacitance) indicate that water storage in the perianth plays an important role in maintaining water balance, and thus, cell turgor (Chapotin *et al.*, 2003; Roddy *et al.*, 2013, 2018, 2019). The strategies that flowers use to maintain water supply are also important for regulating cell turgor. Previous studies have demonstrated that in flowers where water is transported through the xylem, water is lost at high rates through floral transpiration, and this organ maintains water balance through high hydraulic conductance (Roddy *et al.*, 2016, 2019). The water transport pathway in flowers is unclear, and there is little direct evidence supporting this pathway, especially in non-woody plants (Chapotin *et al.*, 2003; Roddy *et al.*, 2016, 2019, McMann *et al.*, 2022). The mechanisms that underlie flower water supply can be detected by measuring perianth water loss, and the perianth desiccation avoidance can be calculated from water loss curves (Hao *et al.*, 2010).

Floral longevity is also related to nutrient metabolic processes. Differences in the abundance of major metabolites in flowers can be determined by comparative metabolite analysis. This analysis has been used in different flower organs and at different stages of flower development, to illuminate physiological processes and metabolic pathways that regulate flowering time (Gawarecka and Ahn, 2021), the formation of flower organs (Park *et al.*, 2018; Li *et al.*, 2020), and flower stress responses (Paul *et al.*, 2022). Thus, metabolite profiling may offer important information on nutrient use during the flowering process. Sugar is vitally important in flower metabolism, and significant changes in sugar content have been observed in the development of many relatively short-lived flowers. For example, total sugar content in three species with relatively short flower lifespans (i.e. *Narcissus tazetta* and *Ranunculus asiaticus*, which both have flower lifespans of 5 d; and *Consolida ajaci*, which has a flower lifespan of 4 d) gradually increases and then decreases during floral development and senescence (Waseem and Inayatullah, 2011a, b; Gul *et al.*, 2015). Another effective way to understand resource investment strategies of flowers is to establish an economics spectrum. A positive relationship between floral longevity and dry mass per unit area of the perianth has been found in one orchid species (Zhang *et al.*, 2017), suggesting that maintenance of long-lived orchid flowers is associated with higher investment in flower tissues. In addition, reutilization of key nutrients is an important mechanism by which flowers improve nutrient utilization efficiency. Many flowers have a strong ability to reuse nitrogen and phosphorus in the perianth (Verlinden, 2003; Chapin and Jones, 2007, 2009; Jones, 2013). This resorption is accompanied by a decline of perianth mass. In all orchids, flowers at the end of their lifespan gradually lose dry weight instead of undergoing abscission. This dramatic loss of dry mass at flower withering may indicate that orchid flowers have a strong ability to reutilize nutrients.

Different flower structural units fulfil distinct functions, e.g. sepals or calyxes protect flowers, petals or corolla attract pollinators (Galen, 1999). In orchids, the sepals are as colourful as petals. Both sepals and petals can protect the flower and attract pollinators. In addition to structural adaptations, different structural components of flowers may fulfil diverse functions during development through metabolic adaptations (Guo *et al.*, 2009; Xin *et al.*, 2019). Interestingly, orchid flowers adapt to diverse environments through the diversification (e.g. shape, colour) of the labellum (lip), petals, and sepals (Cozzolino and Widmer, 2005; Mondragon-Palomino and Theissen, 2008). Several studies have elucidated the molecular mechanisms underlying the development of diverse perianth structural units (Hsu *et al.*, 2015, 2021) and the functional cooperation between labellum, petals, and sepals during pollination (Fenster *et al.*, 2004; Cozzolino and Widmer, 2005; Breitkopf *et al.*, 2015). However, we still know little about the anatomical and physiological differences between labella, sepals, and petals of an orchid flower and how these differences are related to floral longevity.

*Dendrobium* is one of the most species-rich genera in *Orchidaceae*. The members of this genera are all epiphytic, and a large number of cultivars with large and long-lived flowers have been bred. This provides a good system for us to study the maintenance mechanism of long-lived flowers. In the present study, we hypothesize that (i) long flower lifespan is related to higher resource investment and efficient water and nutrient utilization in *Dendrobium* species; and that (ii) water and nutrient utilization are specialized in sepals, petals, and the labella of long-lived flowers of orchids, i.e. labella consume more water and nutrients to extend flower display, while sepals and petals compensate for this consumption through a more conservative strategy. To test these hypotheses, we established which functional and physiological traits were correlated with flower longevity in 23 *Dendrobium* species or cultivars, and compared the differences in functional traits among the sepals, petals, and labella of mature flowers. In addition, we determined whether complementary water and nutrient use strategies in different flower structural units during flower development were responsible for flower longevity in one *Dendrobium* cultivar with long-lived flowers.

## Materials and methods

### Plant materials and experimental design

**Fig. 1.**
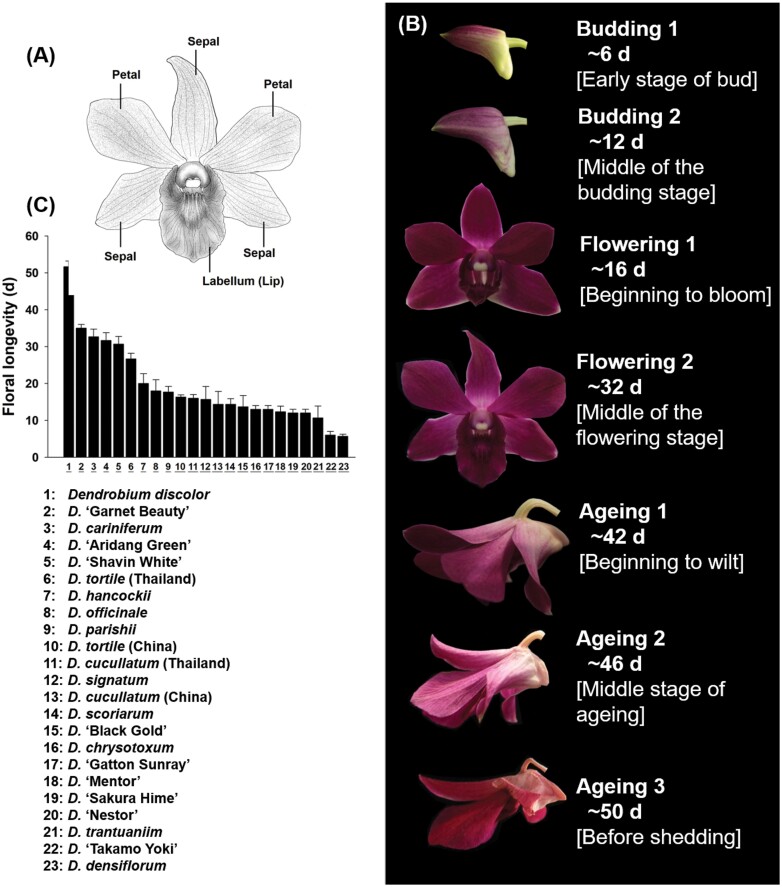
Details of *Dendrobium* flower and cultivars. (A). A diagram of a *Dendrobium* flower showing sepals, petals, and labella. (B) Flower developmental stages of *Dendrobium* ‘Garnet Beauty’. (C) *Dendrobium* species and cultivars in order of floral longevity from the longest to the shortest.

To identify the factors that may be closely related to flower longevity in *Dendrobium*, we compared the flower lifespans of 14 native species and nine cultivars, and the functional traits and physiological properties of their sepals, petals, and labella (Fig. 1; Supplementary Table S1). For this purpose, we measured anatomical traits in each plant, including vein density, epidermis thickness, perianth thickness, and parenchyma cell size of the sepal and petal. We also measured dry mass per unit area of the perianth, biomechanical strength and osmotic potential of sepal, petal and labellum, for each of these species and cultivars. All tested species or cultivars were grown in a greenhouse for at least 3 years at the Flower Research Institute, Guangxi Academy of Agricultural Sciences, Guangxi, China. Growth conditions were as follows: air temperature ranged from 25–30 °C, relative humidity between 70–90%, and 40% full sunlight achieved with a shading net. Experimental materials were planted in 1.5 l plastic pots with a bark mixture, and watered every week to maintain a substrate water content of 65–75%.

We examined the water loss rate, nutrient content and resorption efficiency, and performed a metabolome analysis of one cultivar, *Dendrobium* ‘Garnet Beauty’ across seven developmental stages. We have named these stages according to previous studies on flower development (Gul *et al.*, 2015; Ma *et al.*, 2018): ‘budding 1’ refers to the early budding stage, when the bud appears for about 6 d; ‘budding 2’ refers to the stage between ‘budding 1’ and the end of budding; ‘flowering 1’ refers to the stage when flowers just open; ‘flowering 2’ is the stage between ‘flowering 1’ and the end of blooming; ‘ageing 1’ is the stage when the flower begins to wilt; ‘ageing 2’ refers the stage between ‘ageing 1’ and ‘ageing 3’; ‘ageing 3’ is the stage at which the flower begins to shed (Fig. 1B). Because previous studies found that micro-nutrients are remobilized in flowers only during pollination-induced senescence (Verlinden, 2003; Chapin and Jones, 2007, 2009), we measured nutrient utilization following pollination-induced senescence in *Dendrobium* ‘Garnet Beauty’.

To monitor flower lifespan, 10 floral buds from at least three individuals with racemose inflorescence for each species or cultivar were marked. Floral longevity was calculated as the time interval from a fully opened flower to the closing of the labellum, or senescence of the perianth. For example, the time from stage of flowering 1 to ageing 1 was determined as flower lifespan of *Dendrobium* ‘Garnet Beauty’ (Fig. 1). Observations were carried out every 3 d.

### Anatomical and morphological observation of perianth

To determine the vein density of sepals and petals, pigments of perianths were first removed by soaking the perianth in a FAA (formalin: acetic acid: alcohol: distilled water, 10:5:50:35 v/v) solution for ~30 d or until samples were nearly transparent. We then photographed a section of each sample away from the main vein and the margin of the perianth at 5 × magnification, with a digital camera mounted on a Leica DM2500 microscope (Leica Microsystems Vertrieb GmbH, Germany). Vein lengths were determined from digital images via the Image J program, and values for vein density were expressed as vein length per unit area. Vein density was examined in six perianth replicates per experimental group.

To measure the thickness of cuticle, epidermis, the whole perianth, and the size of parenchyma cells, we made free-hand transverse sections of perianths using razor blades. These perianth sections were then photographed with a Leica DM2500 microscope. Thicknesses of the cuticle and epidermis were photographed at 20 × magnification, and the thickness of whole sepals or petals, and the size of parenchyma cells of the perianth were photographed at 10 × magnification (Supplementary Fig. S1). The size of parenchyma cells was represented by the cell area in perianth transverse sections. Six perianths were measured per experimental group.

To calculate dry mass per unit area of each structural component of perianths, the area was scanned with a leaf area meter (Li-Cor, 3100A, USA), and the dry weight was measured after the perianth was oven dried at 60 °C for 48 h. Subsequently, dry mass per unit area was calculated as dry weight divided by area.

### Water loss measurement in the perianth

The rate of water loss in sepals, petals and labella of *Dendrobium* ‘Garnet Beauty’ at two budding and two flowering stages was measured as follows. Flowers or buds were collected between 17.00–18.00 h, flowers at different developmental stages were collected at the same time and location. Individuals of similar size were collected and transported to the laboratory using an ice box. Flowers at different developmental stages were placed in distilled water, with recut pedicels interacting with water overnight. Each structural component of perianths at different flowering stages was measured for saturated fresh weight (SFW), then placed in a clean tray to let them lose water in the laboratory with an air conditioner unit maintaining a constant temperature of 25 ± 3 °C and the relative humidity of 70–80%. Fresh weight (FW) was measured periodically for 450 h on a digital balance and then perianths were oven-dried at 60 °C for 48 h to obtain dry weight (DW). Relative water content (RWC) was calculated at each time point as follows: (FW—DW)/(SFW—DW) × 100. Water loss was reflected by the curve of relative water content over time. The time required for a saturated perianth to drop to a RWC of 70% (T_70_), the threshold for physiological damage, was determined from the curve (Hao *et al.*, 2010). Relative water content was also recorded in the naturally ageing process of *Dendrobium* ‘Garnet Beauty’.

### Nutrient content and resorption in the perianth

Mature and senescent sepals, petals, and labella of *Dendrobium* ‘Garnet Beauty’ were dried at 60 °C for 48 h to a constant mass weight. Samples were then ground into a powder and passed through a 100-mesh sieve. The content of total carbon (C) and nitrogen (N) was determined with an Isotope Ratio Mass Spectrometer (DELTA V Advantage, Thermo Fisher Scientific, Germany), and the total phosphorus (P) content was measured by inductively coupled plasma atomic emission spectroscopy (ICP-AES: Thermo Jarrell Ash Corporation, USA) after samples were digested in concentrated HNO_3_-HClO_4_ and HCl.

N and P resorption efficiency was calculated using two methods. In the first method, both were calculated as follows: (1—n_s_/n_m_) × 100, where n_s_ and n_m_ are the N or P concentrations of senescent and mature perianths, respectively. In the second method, resorption efficiency was also corrected, using a mass loss correction factor (MLCF), which was calculated as (1—n_s_/n_m_ × MLCF) × 100. MLCF was calculated as dry mass of senescent perianth/dry mass of mature perianth (Vergutz *et al.*, 2012).

### Biomechanical strength and osmotic potential of perianth

To test the biomechanical strength of perianths in all species and cultivars, the flowers or buds were collected between 17.00–18.00 h from the plants that had been watered daily. All the samples were brought to the laboratory using an ice box. The force to punch (F_p_) was measured from 23 species or cultivars by a digital force gauge (ZQ-990A, Dongguan Zhiqu Precision Instrument, China). According to the method described in Onoda *et al.* (2011), F_p_ was calculated as: F_max_/punch rod circumference, F_max_ was the maximum force determined by the digital force gauge for each perianth, and the diameter of the flat-end punch rod was 0.5 mm.

The osmotic potential of each structural component of perianths was measured with a vapor pressure osmometer (Vapro 5520, Wescor, USA; Bartlett *et al.*, 2012). One 28 mm^2^ disc-shaped sample was collected from sepals, petals, and labella of each flower with a 6 mm diameter borer. Samples were wrapped in aluminium foil and frozen in liquid nitrogen for at least 5 min. Immediately after, each sample was punctured about 10 times using a sharp-tipped forcep, with exposure time in air less than 30 s after removal from liquid nitrogen. The equilibrium solute concentration value (c_0_) of the disc-shaped sample was recorded from the osmometer when the difference between consecutive measurements fell below 5 mmol kg^–1^. This value was converted into osmotic potential values using the van’t Hoff equation (Bartlett *et al.*, 2012), which relates solute concentration to vapor pressure. The osmotic potential was calculated as follows: 2.5/1000 × c_0_.

### Metabolome analysis of perianth

For primary metabolome analysis, sepals, petals, and labella sampled from different developmental and ageing stages of *Dendrobium* ‘Garnet Beauty’ were frozen in liquid nitrogen immediately after collection. Before sample extraction, freeze-dried materials were ground into powder by a grinding miller (MM 400, Retsch, Germany) at 30 Hz for 1.5 min. Subsequently, 100 mg of powdered sample was dissolved in 1.0 ml of 70% methanol extraction solution and stored at 4 °C for 24 h. Before LC-MS analysis, the extracts were absorbed onto a CNWBOND Carbon-GCB SPE Cartridge (ANPEL, Shanghai, China) and filtered with an SCAA-104 membrane (0.22 μm, ANPEL, China).

Metabolites were analysed using a liquid chromatography-electrospray ionization-tandem mass spectrometry system (LC-ESI-MS; LC, Shim-pack UFLC Shimadzu CBM30A system, Japan; ESI, MS, Applied Biosystems 6500 QTRAP, USA). Chromatographic separation was executed by an ACQUITY UPLC HSS T3 C18 (1.8 mm, 2.1 mm × 100 mm; Waters, USA). The effluent was alternatively connected to an ESI-triple quadrupole-linear ion trap (QTRAP)-MS. Linear ion trap (LIT) and triple quadrupole (QQQ) scans were acquired on an API 6500 QTRAP LC/MS/MS system equipped with an ESI turbo ion-spray interface operating in positive ion mode and controlled by Analyst 1.6 software (AB Sciex, USA). Metabolite identification was based on the parametric values (m/z data, retention time, and fragmentation partners) and compared with a self-built database (MetaWare) for annotation results (http://www.metware.cn/).

### Statistical analysis

Analysis of variance (ANOVA) and independent-sample *t*-tests were performed for each species and at each developmental stage with SPSS 20.0, and Tukey’s multiple comparison tests was used at the level of α =0.05 to determine whether significant differences existed between different structural units of perianths. Metabolite differences between pairwise comparisons were analysed by partial least squares-discriminant analysis (OPLS-DA). The relative importance of each metabolite to the OPLS-DA model was evaluated using the variable importance in projection (VIP). Metabolites with VIP ≥1 and fold change ≥1 or fold change ≤–1 were identified as being differentially accumulated.

## Results

### Variation and correlation of floral longevity, physiology, and anatomy in *Dendrobium* species and cultivars

**Fig. 2.**
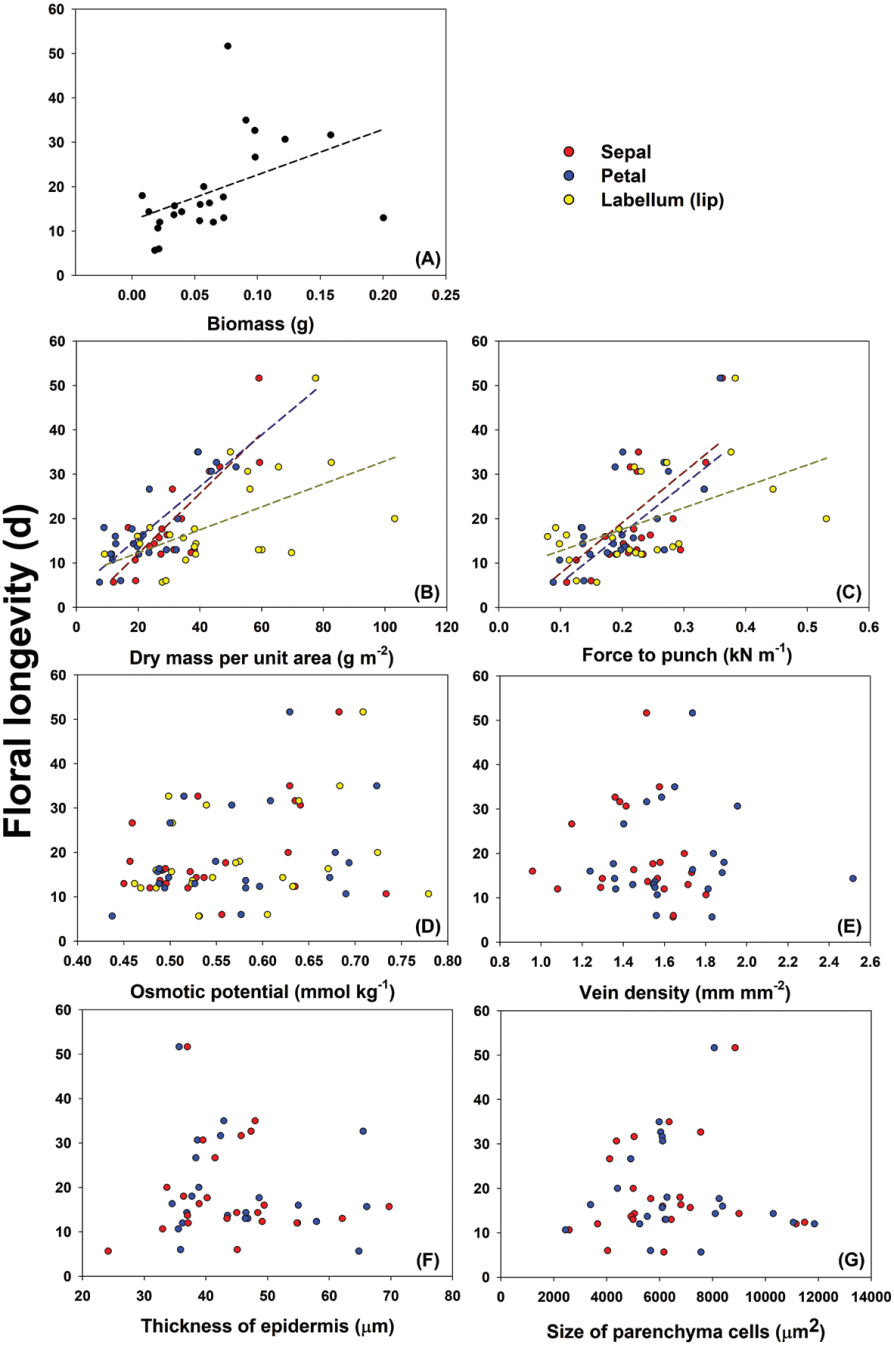
Correlations between floral longevity and anatomical or physiological traits of sepals, petals, and labella. (A) Correlation of total biomass of sepal, petal and the labella with floral longevity (*r*=0.449, *P*<0.05). (B) The relationship between floral longevity and dry mass per unit area of sepals (*r*=0.817, *P*<0.001), petals (*r*=0.889, *P*<0.001), and labellum (*r*=0.547, *P*<0.01). (C) The relationship between floral longevity and force to punch of sepals (*r*=0.670, *P*<0.001), petals (*r*=0.719, *P*<0.001), and labella (*r*=0.518, *P*<0.05). The relationship between floral longevity and osmotic potential (D); vein density (E); thickness of epidermis (F); and size of parenchyma cells (G).

Floral longevity among *Dendrobium* species or cultivars differed greatly, with the maximum flower longevity eight times that of the shortest (Fig. 1C). Floral longevity was positively correlated with total biomass of all perianths, as well as the dry mass per unit area and force to punch of each structural component of the perianth (Fig. 2A-C). However, floral longevity was not correlated with osmotic potential of any perianth component (Fig. 2D). When these relationships for species and cultivars were analysed separately, the same patterns remained (Supplementary Fig. S2A-F).

**Fig. 3.**
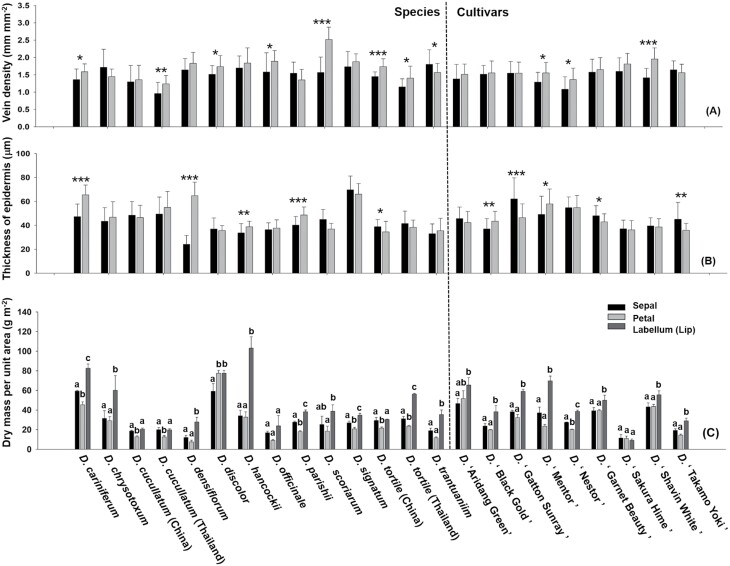
Perianth differences across *Dendrobium*. Differences in vein density (A); thickness of epidermis (B); and dry mass per unit area of perianths from different species and cultivars (C). Statistical differences between the sepals and petals of each species or cultivars were determined with independent-sample *t*-tests (*P<0.05; **P<0.01, ***P<0.001). Different letters above bars indicate significant differences between sepals, petals, and labella for each species or cultivar (*P*<0.05, based on ANOVA, followed by Tukey’s post-hoc tests for comparison).

Anatomical and physiological traits varied between the structural components of perianths in *Dendrobium* species. In 12 out of 14 *Dendrobium* species tested, the dry mass per unit area was lowest for the petals and highest for the labella. For cultivars, the dry mass per unit area was also highest for the labella, except for *Dendrobium* ‘Sakura Hime’, in which dry mass per unit area showed no significant difference between perianth components (Fig. 3C). The biomechanical strength of different components of perianths varied among *Dendrobium* species and cultivars. In nine of these, the toughest flower part was the sepals, whereas in eight species or cultivars it was the labella (Supplementary Fig. S3C). In 19 *Dendrobium* species or cultivars tested, the osmotic potential did not differ significantly between sepals, petals and labella. However, in *D. discolor*, *D. hancockii*, and *D. officinale*, the osmotic potential was highest in the petals; furthermore, in *D. parishii* and *D.* ‘Nestor’, osmotic potential was highest in the labella (Supplementary Fig. S3B).

Vein density, epidermis thickness, the size of parenchyma cells, and perianth area were not correlated with floral longevity, regardless of whether species and varieties were analysed together (Fig. 2D-G; Supplementary Table S2), or whether they were analysed separately (Supplementary Fig. S4A-F). The vein density of petals in eight *Dendrobium* species was higher than that of sepals (Fig. 3A). In six out of 10 *Dendrobium* species or cultivars, the epidermis of petals was thicker than that of sepals. Furthermore, in seven out of 10 *Dendrobium* species or cultivars, parenchyma cells were larger in petals than in sepals (Supplementary Fig. S3A).

In sepals, epidermis thickness positively correlated with that of their perianth thickness, whereas in petals, there was no significant relationship between epidermis thickness and perianth thickness. The thickness of perianths positively correlated with flower biomass and dry mass per unit area of perianth for both sepals and petals (Supplementary Table S2). All morphological and physiological traits of sepals and petals were correlated, except for epidermis thickness (Supplementary Fig. S5A-G). All physiological traits of the labella positively correlated with those of sepals or petals (Supplementary Fig. S5H-J).

### Functional and physiological traits during development of a long-lived flower

**Fig. 4.**
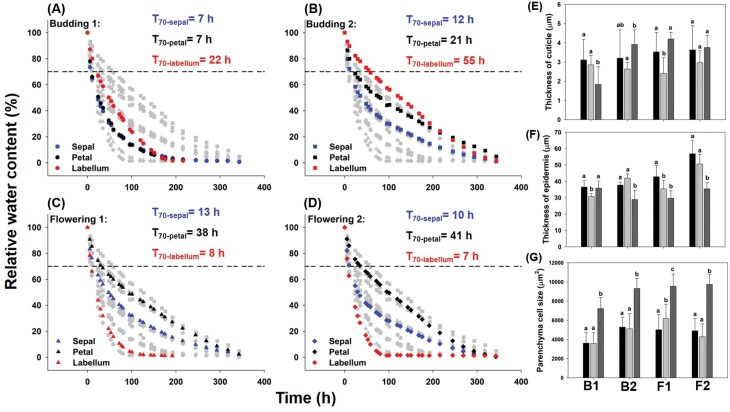
Changes in water utilization and anatomical traits during development and senescence of flowers in *Dendrobium* ‘Garnet Beauty’. (A-D) Water loss rate of perianth structural units at different developmental stages; (E) cuticle thickness; (F) epidermis thickness; and (G) parenchyma cell size in sepals, petals, and labella during flower development. T_70_ represents the time required for a saturated perianth to drop to relative water content of 70%. Different letters above bars indicate significant differences between sepals, petals and labella at each developmental stage (*P*<0.05, based on ANOVA, followed by Tukey’s post-hoc tests for comparison). B1: budding stage 1, B2: budding stage 2, F1: flowering stage 1, F2: flowering stage 2.

The rate of water loss in different structural units of perianths at different developmental stages was varied. Water loss was most rapid in budding stage 1, when the time required for a water-saturated perianth to drop to relative water content of 70% (T_70_) was the lowest for all units of perianths. The T_70_ of petals increased with flower development. Water loss was faster from the labella than from sepals and petals at flowering stages (Fig. 4A-D). These differences were in line with rates of water loss during developmental ageing (Supplementary Fig. S6C). Water loss from sepals was relatively constant, compared with that of petals and labella during developmental stages.

Anatomical features varied between the structural units of perianths during flower development. Specifically, when the budding flower emerged at ~6 d (budding stage 1), the cuticle was the thickest in the sepals (Fig. 4E). In addition, the cuticle of the labella developed faster than that of the petals; thus, when flower buds were 12 d old (budding stage 2) and when buds just opened (flowering stage 1), the cuticle was thicker on the labella than on the petals. When flowers became mature, cuticle thickness did not differ significantly between sepals, petals, and labella (*P*>0.05; Fig. 4E). During flower development, the epidermis was the thickest in the sepals (Fig. 4F). Throughout flower development, parenchyma cells were the largest in the labella (Fig. 4G). When the flower bud was 12-day-old, vein density was higher in petals than in sepals, but no significant difference was found between these two units after this stage (*P*>0.05; Supplementary Fig. S6A). The petals were thicker than sepals during flower development, except for the flowering stage 2 (Supplementary Fig. S6A). The biomass, dry mass per unit area, and force to punch, were greater in the labella than in either the sepals or the petals across flower development (Supplementary Fig. S7A, B, D). However, osmotic potential did not differ between sepals, petals, and labella during development (Supplementary Fig. S7C).

**Fig. 5.**
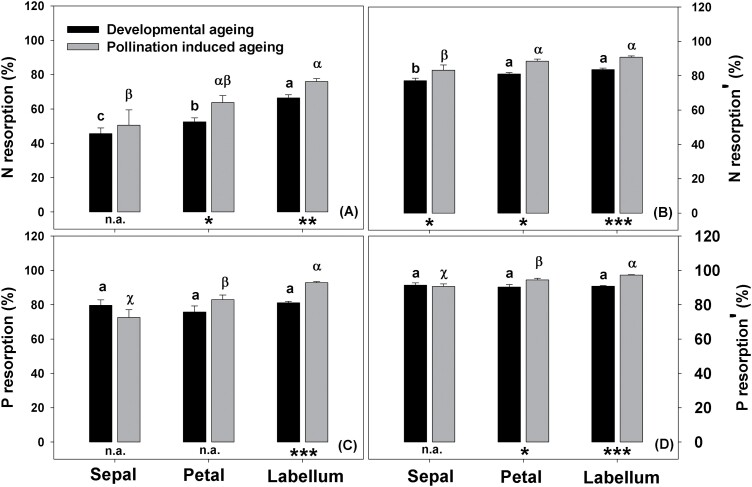
Nutrient resorption efficiency of *Dendrobium*. Nitrogen (A) and phosphorus (C) resorption efficiency; nitrogen (B) and phosphorus (D) resorption efficiency corrected by dry mass loss. Statistical differences between the developmental and pollination-induced ageing for each structural unit of the perianth were determined with independent-sample *t*-tests (*P<0.05; **P<0.01; ***P<0.001). Different letters above bars indicate significant differences between sepals, petals, and labella during developmental ageing and pollination-induced ageing; *P*<0.05, based on ANOVA, followed by Tukey’s post-hoc tests for comparison. N: nitrogen; P: phosphorus.

In mature flowers, carbon content did not differ significantly between sepals, petals, and labella, whereas nitrogen (N) content was relatively higher in labella, and phosphorus (N) content was relatively higher in petals (Supplementary Fig. S8). When calculating N and P resorption efficiency, we corrected for the fact that totally senescent flower dry weight is over 50% less than that of flowers in full bloom (Supplementary Fig. S9A) by using MLCF. During naturally occurring senescence, N resorption efficiency was the highest in labella, and the lowest in sepals. P resorption efficiency did not differ between sepals, petals, and labella (Fig. 5A, C). After correcting for senescent dry weight loss using MLCF, we found higher N and P resorption efficiencies. Specifically, N resorption efficiency was over 70%, and P resorption efficiency was over 90%; furthermore, N resorption efficiency was the same in petals and labella (Fig. 5B, D). During pollination-induced senescence, the N and P resorption efficiencies were the highest in labella and the lowest in sepals (Fig. 5A, C). After MLCF correction, N and P resorption efficiencies were the same for sepals, petals, and labella. Although we found no differences in N and P resorption efficiencies in sepals following natural senescence and pollination-induced senescence, both N and P resorption efficiencies in labella differed between these two types of ageing (Fig. 5B, D).

### Primary metabolite changes during development of a long-lived flower

**Fig. 6.**
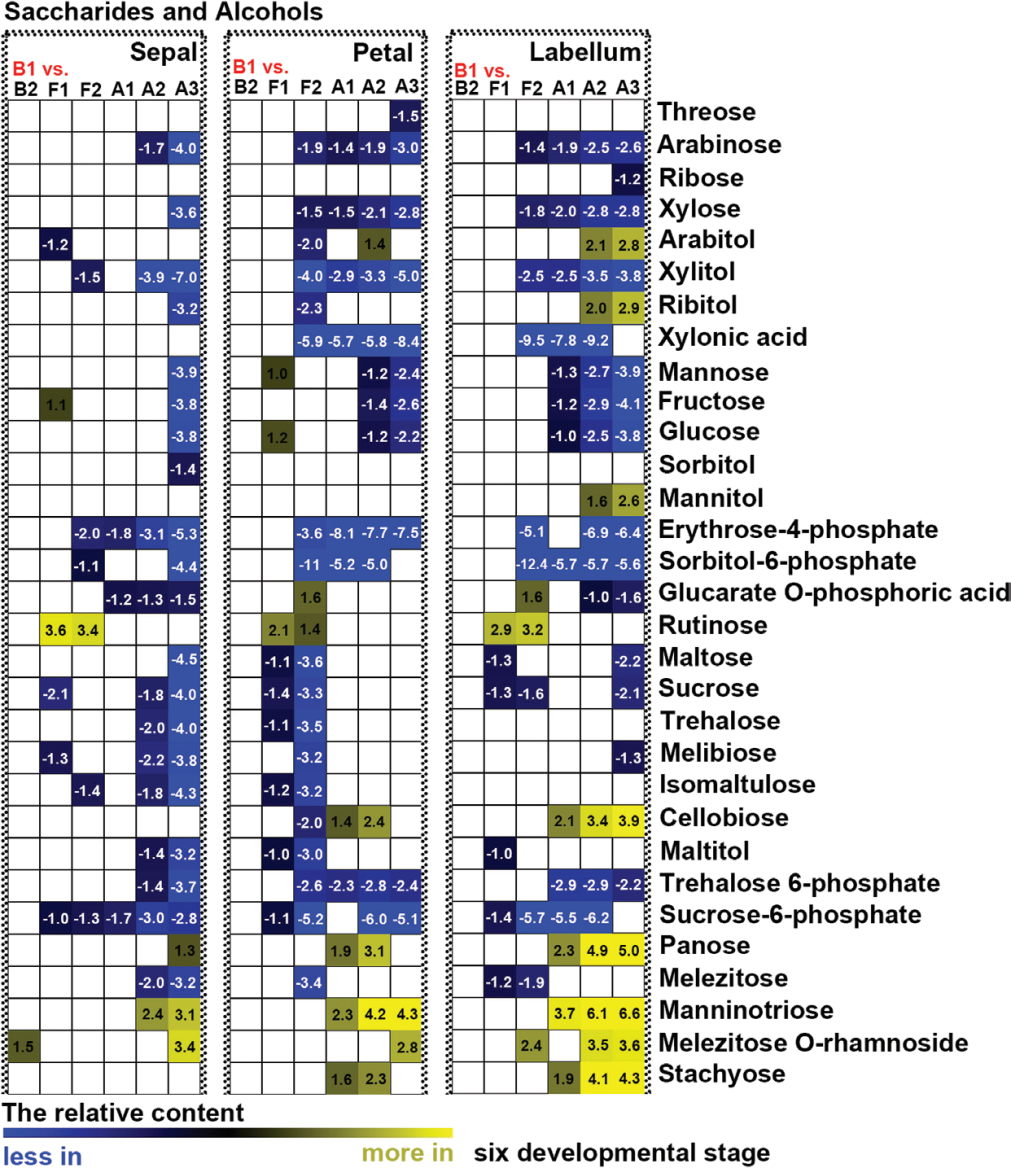
Heat map of relative changes in abundance of saccharides and alcohols during flower development as detected by UPLC-MC. All stages of flower development were compared with budding stage 1 (B1). Numbers on heat map indicate significant fold change between groups under comparison. White in the heat map indicates no significant difference between groups under comparison. B2: budding stage 2, F1: flowering stage 1, F2: flowering stage 2, A1: ageing stage 1, A2: ageing stage 2, A3: ageing stage 3.

**Fig. 7.**
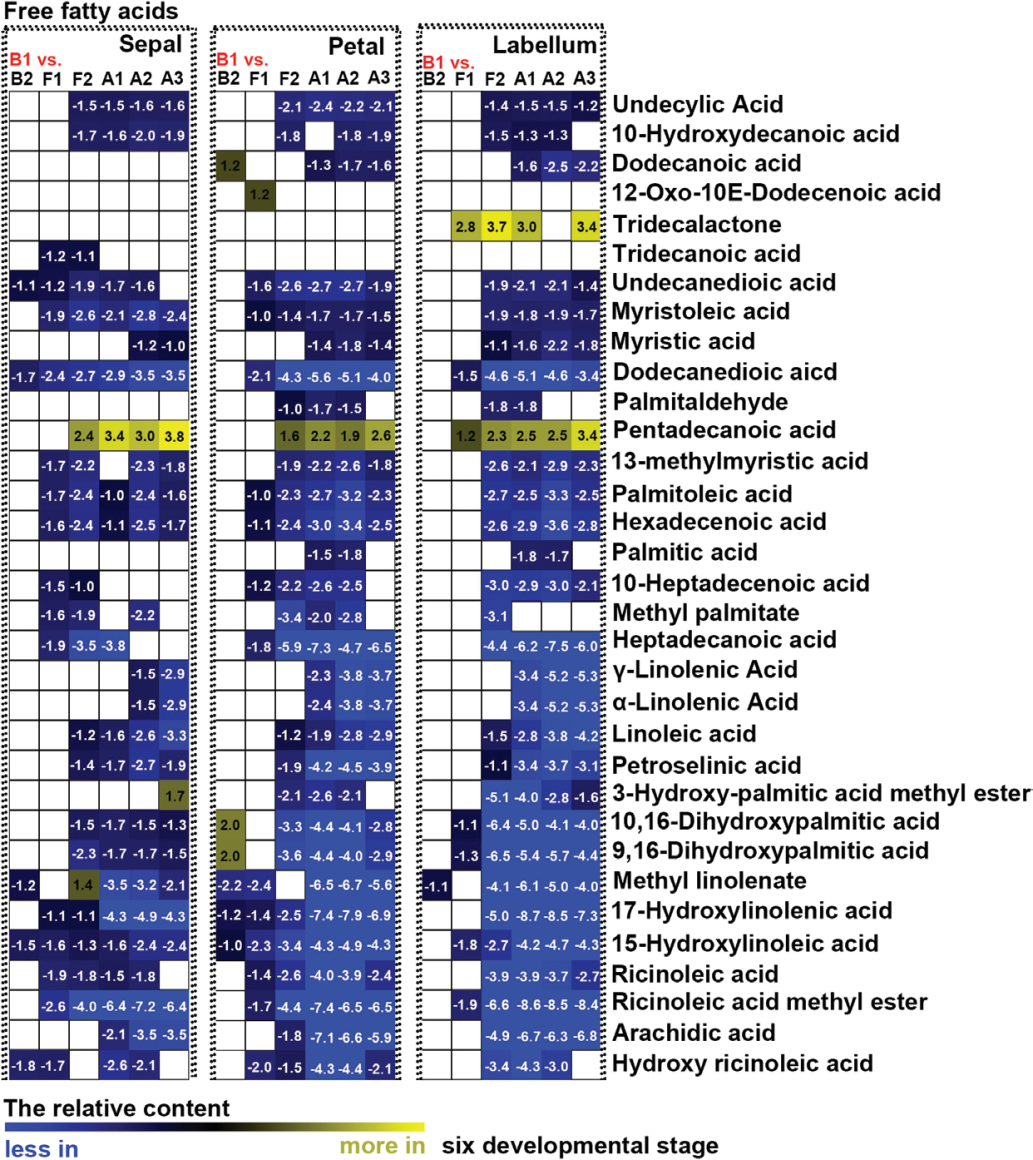
Heat map of relative changes in fatty acid abundance during flower development, as detected by UPLC-MC. All stages of flower development were compared with budding stage 1 (B1). Numbers on heat map indicate significant fold change between groups under comparison. White in the heat map indicates no significant difference between groups under comparison. B2: budding stage 2, F1: flowering stage 1, F2: flowering stage 2, A1: ageing stage 1, A2: ageing stage 2, A3: ageing stage 3.

**Fig. 8.**
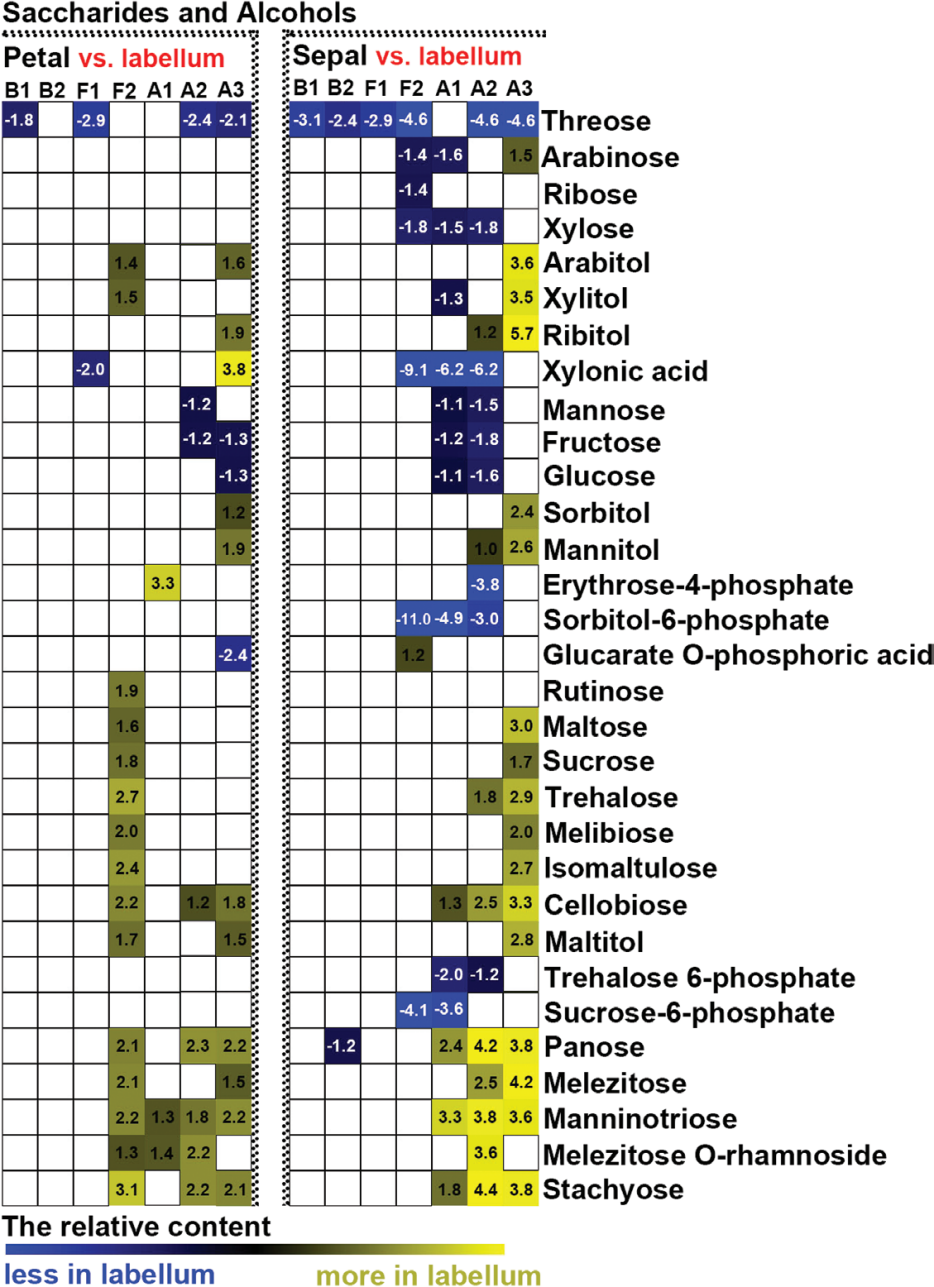
Heat map of relative changes in the abundance of saccharides and alcohols in structural units of the perianth during flower development, as detected by UPLC-MC. Numbers on heat map indicate the fold change between groups under comparison. White indicates no significant difference between groups under comparison. B1: budding stage 1, B2: budding stage 2, F1: flowering stage 1, F2: flowering stage 2, A1: ageing stage 1, A2: ageing stage 2, A3: ageing stage 3.

**Fig. 9.**
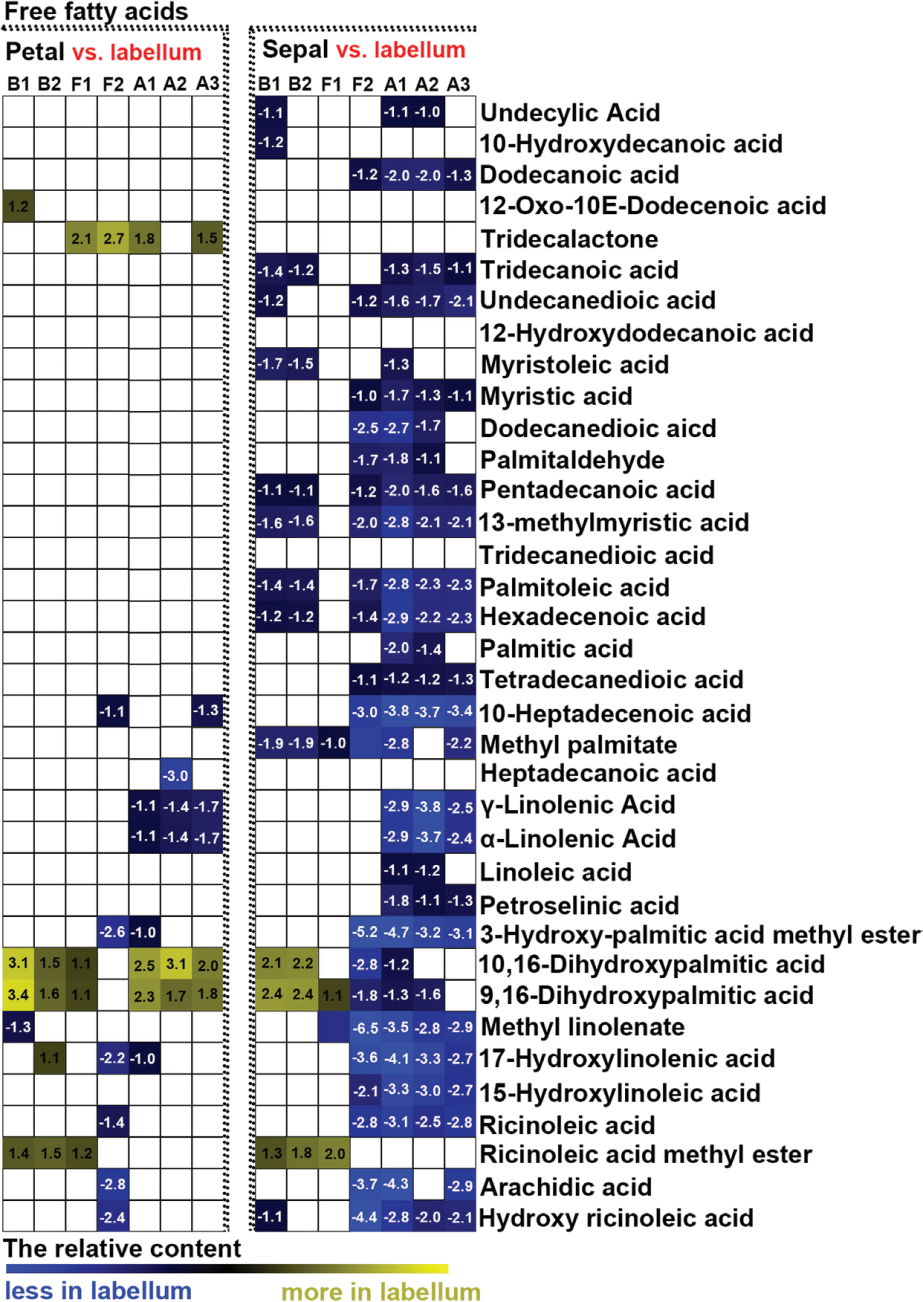
Heat map of relative changes in fatty acid abundance in structural units of the perianth during flower development, as detected by UPLC-MC. Numbers on heat map indicate the fold change between groups under comparison. White indicates no significant difference between groups under comparison. B1: budding stage 1, B2: budding stage 2, F1: flowering stage 1, F2: flowering stage 2, A1: ageing stage 1, A2: ageing stage 2, A3: ageing stage 3.

To detect differences in metabolites during flower development, we compared the changes in relative contents of saccharides, alcohols, amino acids, and free fatty acids between different developmental stages and between different structural components of perianths. Few significant changes in primary metabolites were found in sepals, petals, and labella between budding stages 1 and 2 (Fig. 6, 7; Supplementary Fig. S10). When sepals or petals were compared with labella, few differences in the relative contents of saccharides and alcohols were detected at budding stages 1 or 2 (Fig. 8), whereas relatively higher contents of free fatty acids and amino acids were detected in sepals than in the labella (Fig. 9; Supplementary Fig. S11). At flowering stage 1, which represents the junction between budding and flowering stages, saccharide and alcohol content were constant (Fig. 6), whereas free fatty acid content decreased, and amino acids accumulated, in all structural units of the perianth (Fig. 7; Supplementary Fig. S10). At this same stage, amino acid content was higher in sepals than in the labella (Supplementary Fig. S11). As flowers developed from the budding stage to full bloom, the content of most primary metabolites decreased, although the smallest decrease was observed in free fatty acids in sepals (Fig. 6, 7; Supplementary Fig. S10). At flowering stage 2, the content of saccharides and alcohols was lower in petals than in the labella (Fig. 8). After the flowers wilted, all metabolites showed greater decline, except for some saccharides with larger sugar molecules, which were detected at relatively higher contents in the labella at the ageing stages (Fig. 6). Notably, before flower shedding, the labella had a relatively higher content of saccharides than the sepals (Fig. 8).

## Discussion

Orchids are well-known for their specialized labella and relatively long-lived flowers. Elucidating the mechanisms by which orchids maintain long flower lifespan may provide new insights into ecological adaptation of orchids, and the breeding of new *Dendrobium* cultivars with longer flower lifespan. Here, we found that the flower traits related to water and nutrients are correlated with floral longevity, and *Dendrobium* flowers maintain longevity by increasing resource investment and complementary water and nutrient utilization among the sepal, petals, and labella.

### Flower economics spectrum of *Dendrobium* species

An important approach to understanding of the functional diversity and trait covariation or trade-off of a plant organ (e.g. flower) is to establish an economics spectrum that combines the key structural and physiological traits of the organ. Researchers have built universal economics spectra for leaves and roots (Wright *et al.*, 2004; Onoda *et al.*, 2017; Kong *et al.*, 2019). The leaf economics spectrum reveals strong patterns of trait co-variation, indicated by obvious trade-offs between leaf mass per unit area and leaf lifespan. Furthermore, species with long leaf lifespan have low photosynthetic rates, but high leaf mass per unit area and leaf construction costs (Wright *et al.*, 2004). A feasible scheme of constructing flower economics spectrum was also proposed (Roddy *et al.*, 2021). Although the perianth of the flower is derived from the leaf, the function of the flower is much more complex. The perianth has multiple functions (e.g. attracting pollinators, protecting other plant organs) that are achieved by diverse strategies. In the flower, a positive relationship between dry mass per unit area of perianth (PMA) and floral longevity has been found in *Paphiopedilum* (Zhang *et al.*, 2017), but very little data are available to determine whether this relationship is universal.

In this study, we found that flower longevity in *Dendrobium* species or cultivars was positively correlated with PMA, and that species with higher PMA had a thicker perianth blade and stronger tissue biomechanical strength (Fig. 2B, C; Supplementary Table S2). These findings are consistent with the finding in *Paphiopedilum* orchids (Zhang *et al.*, 2017), and indicate the universal relationship between construction cost and longevity among plant organs. Our results also show that flower lifespan is positively correlated to flower biomass (Fig. 2A). This suggests that flower lifespan is enabled via increased investments in morphological construction and maintenance of physiological functions, confirming our hypothesis, and that compensation for these costs includes increased reproductive success.

We found that in *Dendrobium* species morphological and physiological traits of different structural units of the perianth were significantly positively correlated (Supplementary Fig. S5). The correlations of these morphological traits were stronger between sepals and petals than between sepals and the labella, or petals and the labella. These results are partly different from a previous study that found a stronger correlation between sepals and leaves than between sepals and petals (Roddy *et al.*, 2013). This discrepancy may be related to functional differences amongst perianth structural units. The sepals of orchids are colourful and function similarly to petals, i.e. mainly for pollinator attraction, which differs from green sepals that perform vegetative roles in other plant species. Although the labellum is a specialized petal, it is more versatile than most petals. The labellum not only attracts pollinators, but can also be a landing platform for pollinators, or a cage to trap pollinators (Cozzolino and Widmer, 2005; Schiestl and Schlueter, 2009). Additional evidence of the specificity of the labellum is that the relationship between PMA and flower longevity was different in the labella than in sepals and petals (Fig. 2B). These findings indicate that there is no direct way to compare the labella of orchid flowers to petals of flowers in other plants. More research on irregular flowers may be helpful in establishing a flower economics spectrum.

### Water use strategies in long-lived flowers of *Dendrobium* species

Prolonged flower lifespan increases the probability for successful reproduction, but also increases the costs of maintaining flowers, especially the cost of maintaining water balance (Ashman *et al.*, 1994; Galen, 1999; Teixido and Valladares, 2014; Teixido *et al.*, 2019). Flowers use various mechanisms to maintain both water balance and normal functions during anthesis (Teixido and Valladares, 2014; Roddy *et al.*, 2016, 2018; 2019; Teixido *et al.*, 2019). Our results suggest that the means by which flowers maintain cell turgor varies across *Dendrobium* perianths. For example, the rate of water loss was extremely different among different structural units of the perianth; specifically, the labellum lost water faster at mature stages than at the budding stage, whereas the rate of water loss in petals was lowest at the flowering stage (Fig. 4A-D). The high rate of water loss may help pull water transport required for water supply from the xylem (Zimmerman, 1990; Zimmermann *et al*., 1994). Our findings indicate that different units of the perianth may deploy different water use strategies at different developmental stages. This is consistent with the needs of each structural unit, i.e. the mature labellum needs a larger supply of water, whereas mature petals, with a stronger ability to avoid desiccation, needs relatively less water supply. Thus, in one flower, petals adopt a conservative water use strategy to achieve the display, whereas the labellum uses a risky strategy.

Another approach to maintaining perianth water balance is through water storage. Water storage in parenchyma cells of the perianth itself help maintain cell turgor pressure; indeed, many kinds of flowers have been shown to have high hydraulic capacitance (Chapotin *et al.*, 2003; Roddy *et al.*, 2018, 2019). In *Dendrobium*, the size of parenchyma cells was significantly larger in the labellum than in sepals and petals at all developmental stages (Fig. 4G). Higher water storage in the labellum may meet the need for substantial amounts of water to maintain turgor. Water storage in parenchyma cells of storage organs in flowering plants have been shown to help flowers maintain morphological and physiological normality (Zotz, 1999; Ng and Hew, 2000; Blanchard and Runkle, 2008; Li and Zhang, 2019; Li *et al.*, 2022a). In addition, Zimmerman (1990) found a positive relationship between flower number and pseudobulb number in orchids. All *Dendrobium* species have pseudobulbs as storage organs, therefore further work is needed to explore the relationship between flower function and water supply from pseudobulbs in *Dendrobium* species.

Water and carbon supply are maintained by the vein system, and thus, related to vein density. In leaves, dense veins increase water supply capacity, allowing water to be supplied faster and closer to the evaporation site of the leaf, and increasing the capacity of transpiration and photosynthesis. Consequently, high vein density in leaves allows plants to accumulate more biomass (Sack and Frole, 2006; Brodribb and Feild, 2010). A previous study showed that vein density evolves independently in petals and leaves; specifically, petals have significantly lower vein density than leaves (Roddy *et al.*, 2013). For flowers, lower vein density may indicate areas of the floral display that require low levels of water and carbon. We found that vein density in perianths is not correlated with floral longevity (Fig. 2E), indicating that the high water and carbon needs of flowers are not totally dependent on the vein system for transportation. In addition, we found that vein density varied between sepals and petals in *Dendrobium* species (Fig. 3A), indicating the complexity of water and carbon use strategies in flowers, especially orchid flowers. Further studies will aim to understand water and carbon supply and utilization in *Dendrobium* flowers by focusing on the more refined structures of the vascular bundles, such as thickness of xylem or phloem, plasmodesmata density, and pit membrane area.

### The function of polysaccharides and free fatty acids in long-lived flowers of *Dendrobium* species

Polysaccharides, including extracellular polysaccharides, have been shown to increase the ability of plant organs (e.g. stems, leaves, roots, and flowers) to store water and to buffer water deficits (Nobel *et al.*, 1992; Clifford *et al.*, 2002; Chapotin *et al.*, 2003; Silveira *et al.*, 2020). Previous studies have shown that *Dendrobium* stems accumulate high levels of polysaccharides when they experience environmental and biotic stresses (Huang *et al.*, 2015; Wu *et al.*, 2016; Abdullakasim *et al.*, 2018). In this study, we found that monosaccharides, rather than polysaccharides, were accumulated at higher levels in the labellum than in sepals and petals throughout flower development (Fig. 6, 7). This finding suggests that saccharides are important sources for metabolism in the labellum, and levels of metabolic consumption in the labellum are high. However, more research will be needed to explore the function of polysaccharides in the flowers of orchids, and their contribution to maintaining metabolic function in long-lived flowers.

Fatty acid accumulation has been linked to various biophysical functions, and is known to play a significant role at different developmental stages of plant growth and in defence responses against biotic and abiotic stresses. Previous studies have found that stressed plants markedly accumulate total free fatty acids (Radhakrishnan and Lee, 2013; Gholinezhad and Darvishzadeh, 2021), and early increases in the concentrations of free fatty acids facilitate the adaptability of plants to stress (Sanchez-Martin *et al.*, 2018). We found that all structural units of perianths maintain relatively constant free contents of fatty acid at the budding stage, although the content in sepals was relatively higher than that in petals and the labellum (Fig. 8, 9). This may be because only sepals interact with the environment at budding stages. During the blooming and ageing process, the content of free fatty acids decreased gradually throughout the perianth, although as the perianth aged, fatty acid content in sepals decreased only slightly from the budding stage (Fig. 8). Thus, at all developmental stages, the relative content of free fatty acids was higher in sepals than in petals and the labellum. This implies that sepals play a larger role in defence than do petals or the labellum.

### High nutrient resorption efficiency in long-lived flowers of *Dendrobium* species

Nutrient resorption is an important strategy for the conservation of plant nutrients. Research on flowers has suggested that N and P are highly remobilized nutrients, which are key elements for plant growth. For example, in petunia flowers, N and P contents decrease in naturally senescent flowers, and ~75% of P content, and ~50–60% of N content are reused (Verlinden, 2003; Chapin and Jones, 2009). Our findings for N and P resorption efficiencies in *Dendrobium* flowers were consistent with those in petunia flowers (Fig. 5A-D). Moreover, we found a significant increase in both N and P resorption efficiencies after correction, for dry weight loss in totally senescent flowers, especially in the labellum (Supplementary Fig. S9A). This finding shows that the decrease in dry mass of flowers during senescence leads to an underestimation of nutrient resorption efficiency in flowers. In fact, N and P resorption efficiencies for the flowers of *Dendrobium* species were underestimated by ~30% (Fig. 5B, D). A previous study on leaves has similarly found that leaf mass loss leads to an average nutrient underestimation of 10% when using leaf mass-based concentration (Vergutz *et al.*, 2012).

Previous studies have found that in petunia, both pollinated and unpollinated corollas reuse the macronutrients N, P, and K at similar levels, whereas micro-nutrients are only remobilized in flowers during pollination-induced senescence (Verlinden, 2003; Chapin and Jones, 2007, 2009). In *Dendrobium* species, N and P resorption efficiencies were significantly higher in pollination-induced senescent flowers than in naturally senescent flowers, especially in the labellum (Fig. 5A-D). This indicates the uniqueness of the labellum in nutrient utilization.

**Fig. 10.**
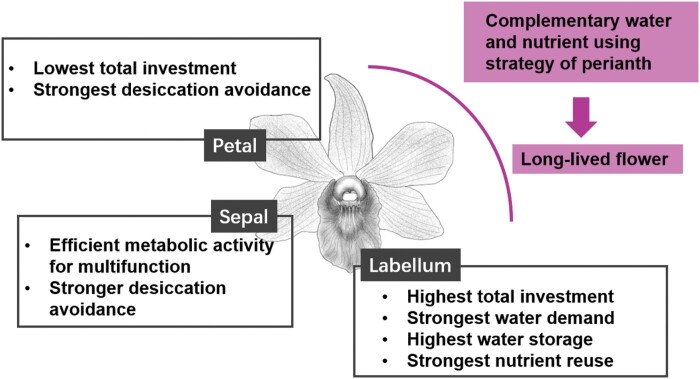
Diagram of complementary water and nutrient utilization strategies of sepals, petal, and labella of long-lived flowers of *Dendrobium* species.

In conclusion, we have shown that flower lifespan differs significantly among both species and cultivars of *Dendrobium.* The long-lived flowers require high construction investment and efficient water and nutrient use. The labellum of long-lived flowers has high nutrient input and metabolic activity with fast water supply and a strong ability to reuse nutrients, while efficient water and metabolic activity indicated by lower investment and stronger desiccation avoidance for sepals and petals reflected their conservative strategies. These complementary water and nutrient utilization strategies among different perianth structural units may help maintain the lifespan of long-lived flowers (Fig. 10). This research provides new insights into the functional diversity of the perianth in one flower, and the anatomical and physiological mechanisms underlying long-lived flowers of orchids. These findings have clear implications for the commercial production of orchids. The extraordinarily showy labellum, which is one reason why orchids continue to be popular in the flower market, consumes the most energy in the flower. Because flower longevity is critical for the flower industry, this likely trade-off between a showy labellum and floral longevity should lead horticulturalists to consider breeding orchid varieties with less showy labella and more showy sepals and/or petals.

## Supplementary data

The following supplementary data are available at *JXB* online.

Table S1. Information for *Dendrobium* species and cultivars.

Table S2. Correlations between anatomical and physiological characteristics in sepals and petals of *Dendrobium*.

Fig. S1. Anatomical examples of petal vein, parenchyma cells, and epidermis and cuticle of *Dendrobium* ‘Garnet Beauty’.

Fig. S2. Correlation between floral longevity and physiological characteristics of sepals, petals, and labella of species or cultivars.

Fig. S3. Physiological difference between sepals, petals, and labella for each species and cultivar.

Fig. S4. Correlation between floral longevity and anatomical traits of sepals, petals, and labella of species or cultivar.

Fig. S5. Correlation between same physiological and anatomical traits of different perianth structural units.

Fig. S6. Anatomical differences in the flowers of *Dendrobium* ‘Garnet Beauty’ during development.

Fig. S7. Physiological differences between sepals, petals, and labella in flowers of *Dendrobium* ‘Garnet Beauty’ during development.

Fig. S8. Nutrient contents of sepals, petals, and labella.

Fig. S9. Dry weight loss of perianth during aging, and correlation of nutrient concentration and resorption efficiency.

Fig. S10. Heat map of relative changes in amino acids during flower development of *Dendrobium* ‘Garnet Beauty’.

Fig. S11. Heat map of difference in amino acids among different structural units of the perianth of *Dendrobium* ‘Garnet Beauty’.

erac479_suppl_supplementary_tables_S1-S2_figures_S1-S11

## Data Availability

The data for all physiological and anatomical traits of *Dendrobium*, water loss rate curve at different developmental stages, and raw data of heat map of amino acids, saccharides and alcohols, and free fatty acids during development are available at Dryad Digital Repository https://doi.org/10.5061/dryad.s4mw6m99f; (Li *et al.*, 2022b).
