# Supplementary material for: Complementary water and nutrient utilization of perianth structural units help maintain long floral lifespan in Dendrobium
Source: J Exp Bot. 2022 Dec 3;74(3):1123–39. doi: 10.1093/jxb/erac479 (PMC9899416; doi:10.1093/jxb/erac479)
Supplement: erac479_suppl_supplementary_tables_S1-S2_figures_S1-S11 [file erac479_suppl_supplementary_tables_s1-s2_figures_s1-s11.pdf]

Complementary water and nutrient utilization of perianths structural units help maintain long flower lifespan in *Dendrobium*

Jia-Wei Li[1], Yi Zhou[1], Zi-Bin Zhang[2], Xue-Qiang Cui[2], Hong-Yan Li[1], Mei-Jing Ou[1], Kun-Fang Cao[1*], Shi-Bao Zhang[3*]

[1] State Key Laboratory for Conservation and Utilization of Subtropical Agro-bioresources, College of Forestry, Guangxi University, Nanning, Guangxi, 530004, China

[2] Flower Research Institute, Guangxi Academy of Agricultural Sciences, Nanning, Guangxi 530007, China

[3] Key Laboratory for Economic Plants and Biotechnology, Kunming Institute of Botany, Chinese Academy of Sciences, Kunming, Yunnan 650201, China

***Correspondence:**

Kun-Fang Cao, kunfangcao@gxu.edu.cn

Shi-Bao Zhang, sbzhang@mail.kib.ac.cn

Manuscript number: JEXBOT/2022/308249

Article Type: Research paper

Subject Code: Plant Environment Interactions

Table S1 Information for species or cultivars.

Name	Classification	Propagation Country
Dendrobium cariniferum	Species	China
D. chrysotoxum	Species	China
D. cucullatum	Species	China
D. cucullatum	Species	Thailand
D. densiflorum	Species	China
D. discolor	Species	China
D. hancockii	Species	China
D. officinale	Species	China
D. parishii	Species	China
D. scoriarum	Species	China
D. signatum	Species	China
D. tortile	Species	China
D. tortile	Species	Thailand
D. trantuaniim	Species	Vietnam
D. ‘Aridang Green’	Cultivars	Thailand
D. ‘ Black Gold ’	Cultivars	Japan
D. ‘ Gatton Sunray ’	Cultivars	Thailand
D. ‘ Mentor ’	Cultivars	Japan
D. ‘ Nestor ’	Cultivars	Japan
D. ‘ Garnet Beauty ’	Cultivars	Thailand
D. ‘ Sakura Hime ’	Cultivars	Japan
D. ‘ Shavin White ’	Cultivars	Thailand
D. ‘ Takamo Yoki ’	Cultivars	Japan

Table S2 Correlations between anatomical and physiological characteristics in sepals or petals.

**Sepal**	Osmatic potential	Force to punch	Dry mass per unit area	Biomass	Sepals or petals area	Vein density	Thickness of sepals or petals	Thickness of epidermis	Size of parenchyma cells
Floral longevity	0.403	0.670**	0.817**	0.533**	0.214	-0.157	0.153	-0.025	0.115
Osmatic potential		-0.018	0.347	0.21	0.012	0.278	-0.132	-0.236	-0.098
Force to punch			0.762**	0.412	0.184	-0.203	0.351	0.184	0.254
Dry mass per unit area				0.717**	0.344	-0.146	0.526*	0.203	0.259
Biomass					0.881**	-0.216	0.625**	0.332	-0.045
Sepals or petals area						-0.26	0.477*	0.358	-0.183
Vein density							-0.076	-0.245	-0.435*
Thickness of sepals or petals								0.434*	0.298
Thickness of epidermis									0.34
**Petal**									
Floral longevity	0.276	0.719**	0.889**	0.501*	0.039	0.047	0.249	-0.177	-0.057
Osmatic potential		-0.053	0.271	0.023	-0.214	0.077	-0.163	-0.444*	-0.275
Force to punch			0.746**	0.411	0.081	0.023	0.294	-0.132	-0.086
Dry mass per unit area				0.650**	0.111	0.042	0.486*	-0.117	-0.02
Biomass					0.766**	-0.226	0.779**	-0.089	-0.054
Sepals or petals area						-0.525*	0.587**	0.034	0.052
Vein density							-0.111	-0.229	-0.197
Thickness of sepals or petals								0.17	0.218
Thickness of epidermis									0.457*

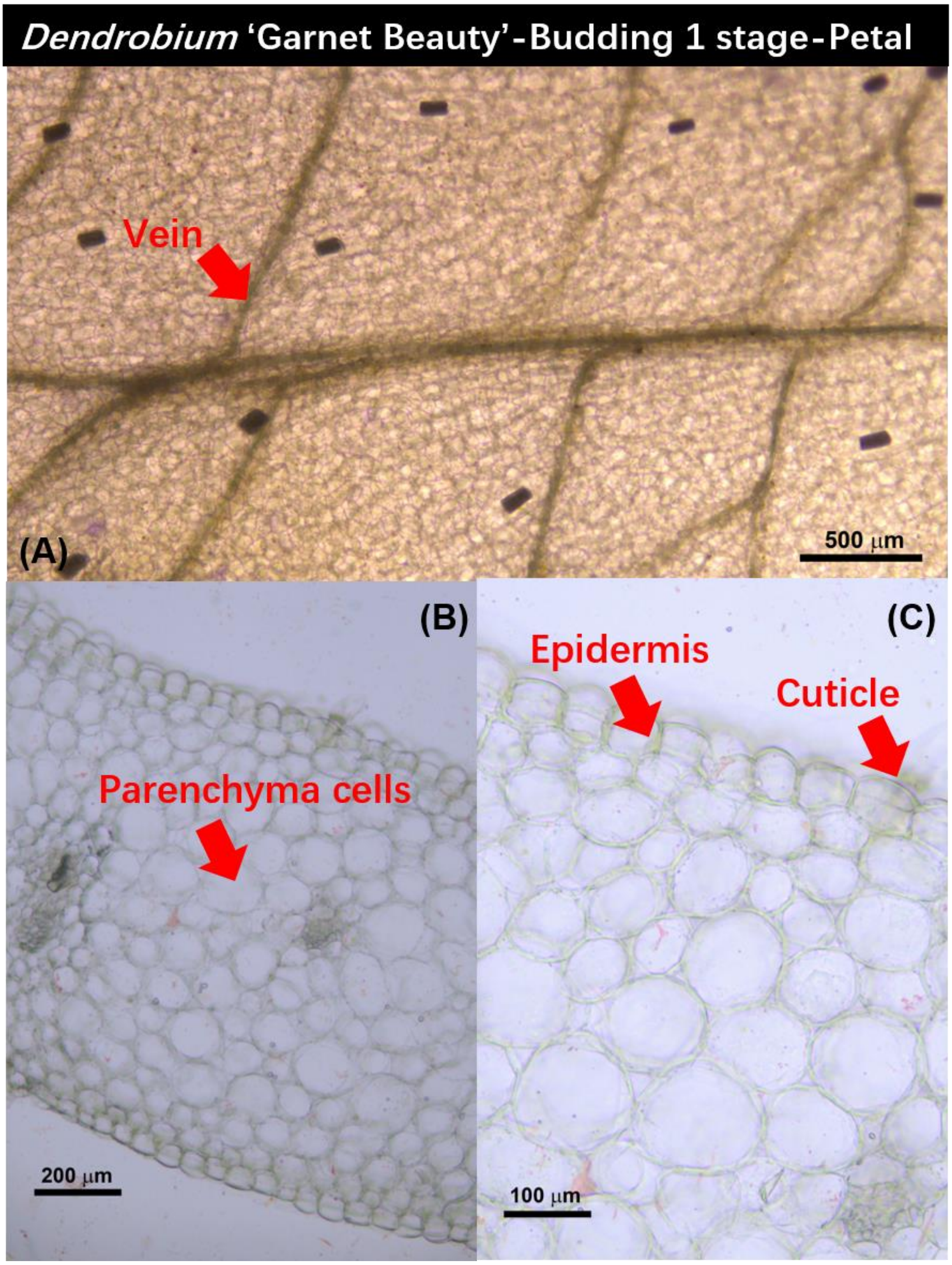

Figure S1 Sample pictures of vein (A), parenchyma cells (B), epidermis and cuticle (C) for petal of *Dendrobium* 'Garnet Beauty' at budding 1 stage.

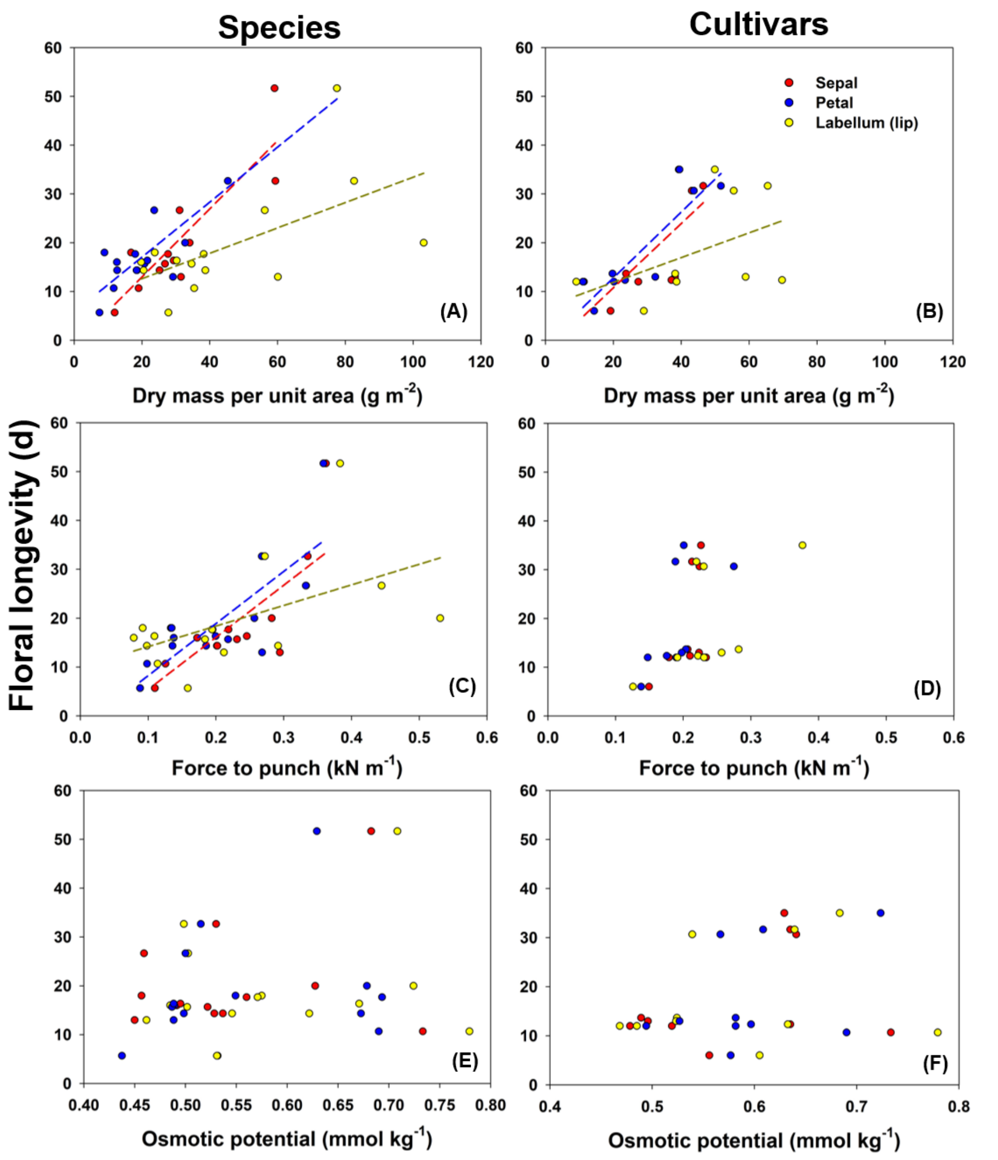

Figure S2 Correlation between floral longevity and anatomical or physiological characteristics of sepals, petals and labella of species or cultivars. (A) Relationship between floral longevity and dry mass per unit area of sepals (r=0.875, P<0.001), petals (r=0.918, P<0.001) and labella (r=0.593, P<0.05) for species; (B) relationship between floral longevity and dry mass per unit area of sepals (r=0.728, P<0.05), petals (r=0.883, P<0.01) and labella (r=0.455, P=0.218) for cultivars; (C) relationship between floral longevity and force to punch of sepals (r=0.767, P<0.001), petals (r=0.785, P<0.001) and labella (r=0.528, P=0.052) for species; (D) relationship between floral longevity and force to punch of sepals, petals and labella for cultivars; (E) relationship between floral longevity and osmotic potential; (F) relationship between floral longevity and osmotic potential.

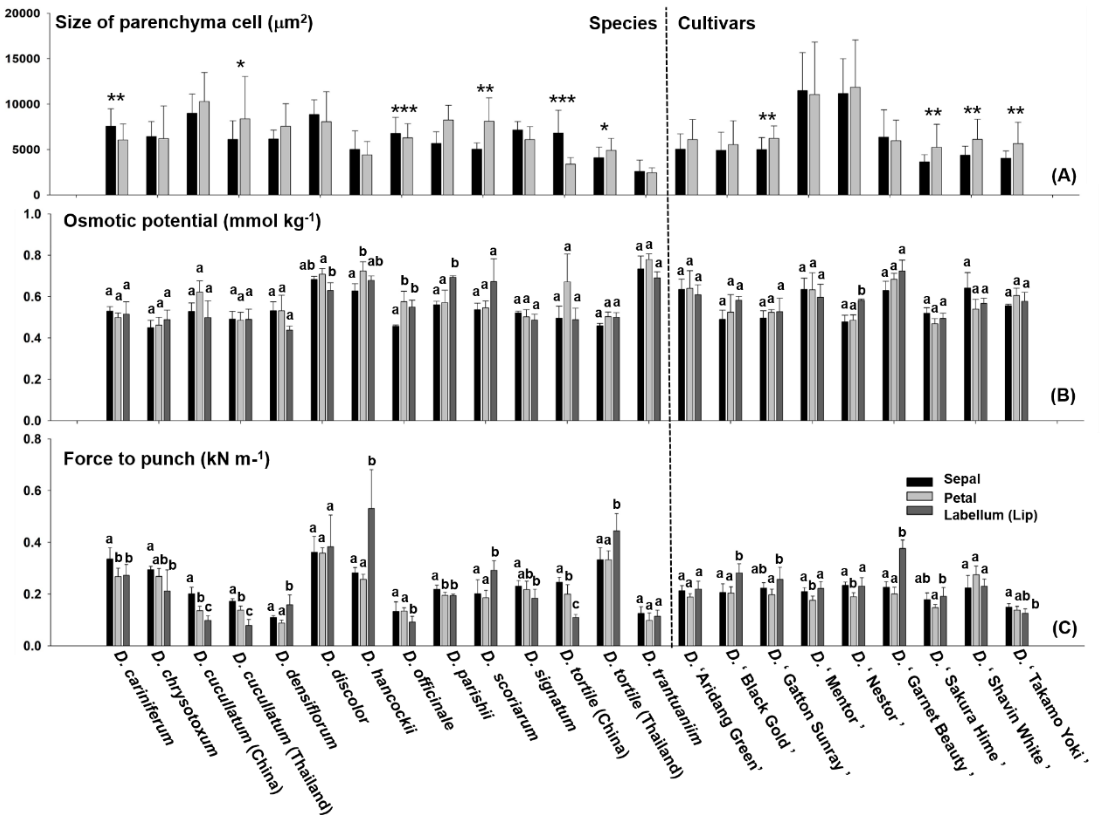

Figure S3 Anatomical and physiological difference between sepals, petals and labella for size of parenchyma cells (A), osmotic potential (B), force to punch (C). Statistical differences (P values) between the sepal and petal for each species or cultivars were determined with independent-sample t tests. $*P < 0.05$; $**P < 0.01$, $***P < 0.001$. Different letters above bars indicate significant differences among sepal, petal and labellum for each species or cultivars. $P < 0.05$, based on ANOVA, followed by Tukey's post hoc tests for comparison.

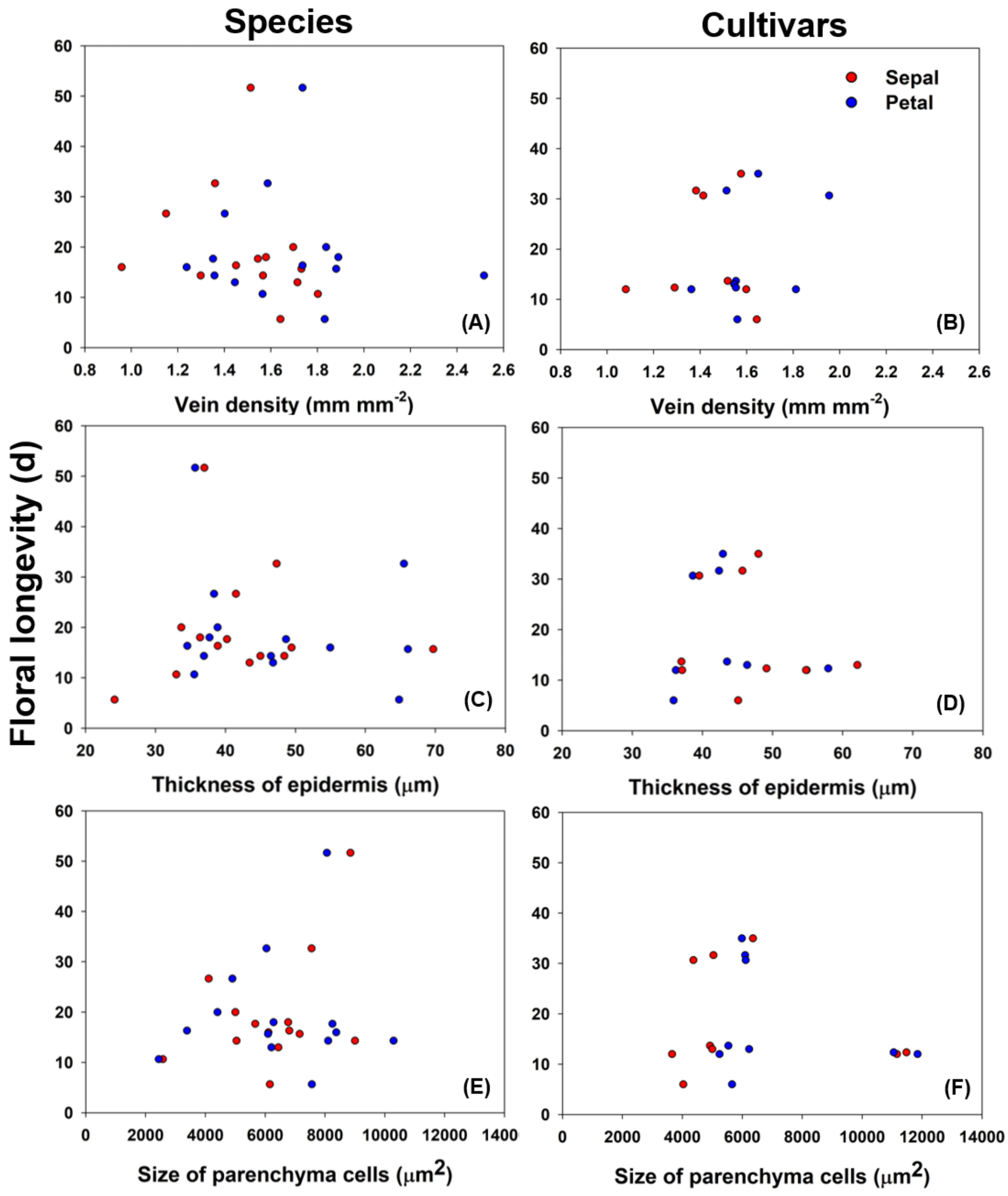

Figure S4 Correlation between floral longevity and anatomical or physiological traits of sepals, petals and labella of species or cultivar. (A) Relationship between floral longevity and vein density among sepals, petals and labella for species; (B) relationship between floral longevity and vein density of sepals, petals and labella for cultivars; (C) relationship between floral longevity and thickness of epidermis of sepals, petals and labella for species; (D) relationship between floral longevity and thickness of epidermis of sepals, petals and labella for cultivars; (E) relationship between floral longevity and size of parenchyma cells; (F) relationship between floral longevity and size of parenchyma cells.

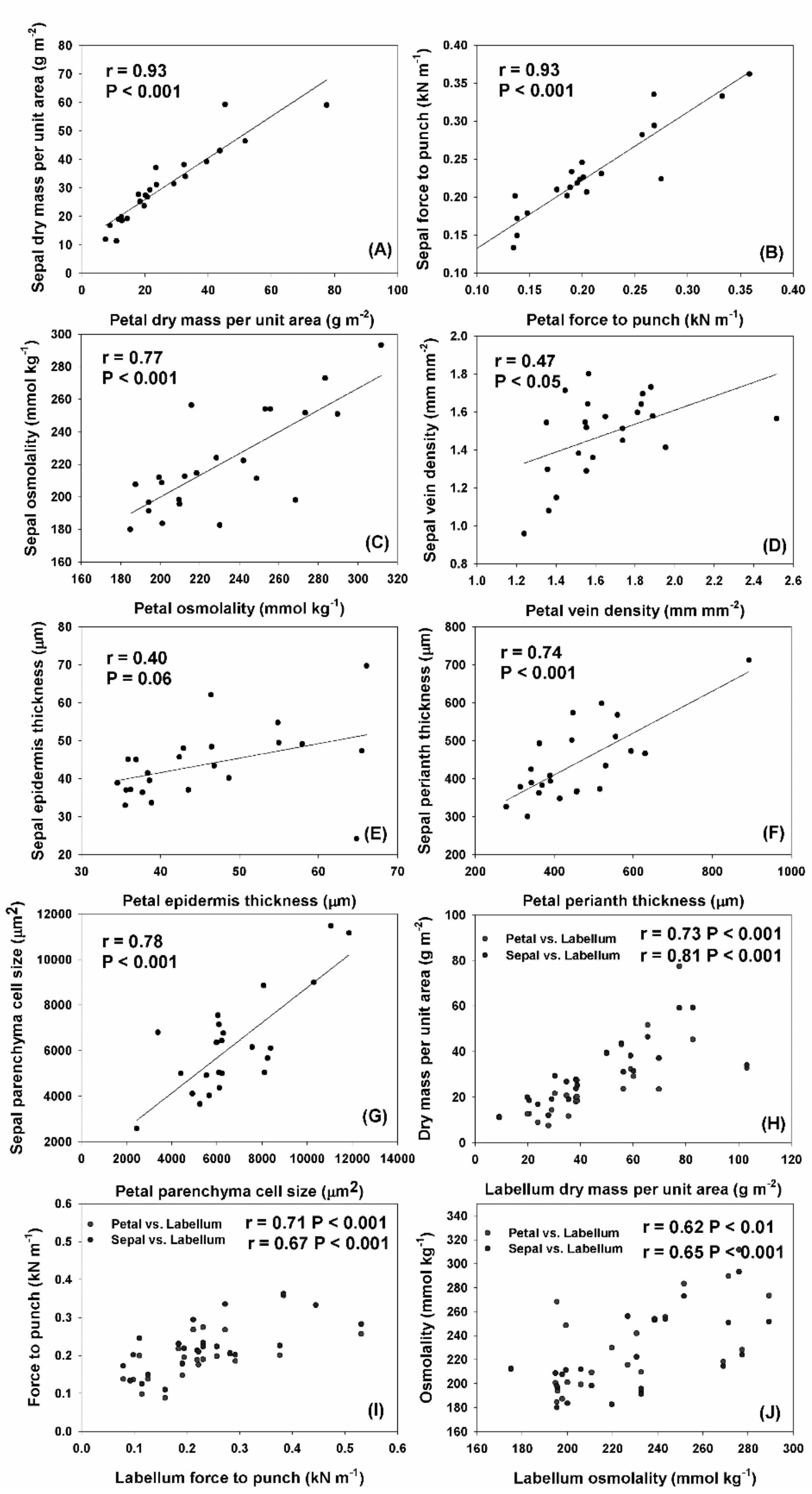

Figure S5 Correlation of same traits between perianth structural units. Correlation of dry mass per unit area between sepals and petals (A), force to punch (B), osmolality (C), vein density (D), epidermis thickness (E), perianth thickness (F), parenchyma cell size (G), correlation of dry mass per unit area (H), force to punch (I), osmolality (J) between petals and labella or between sepals and labella.

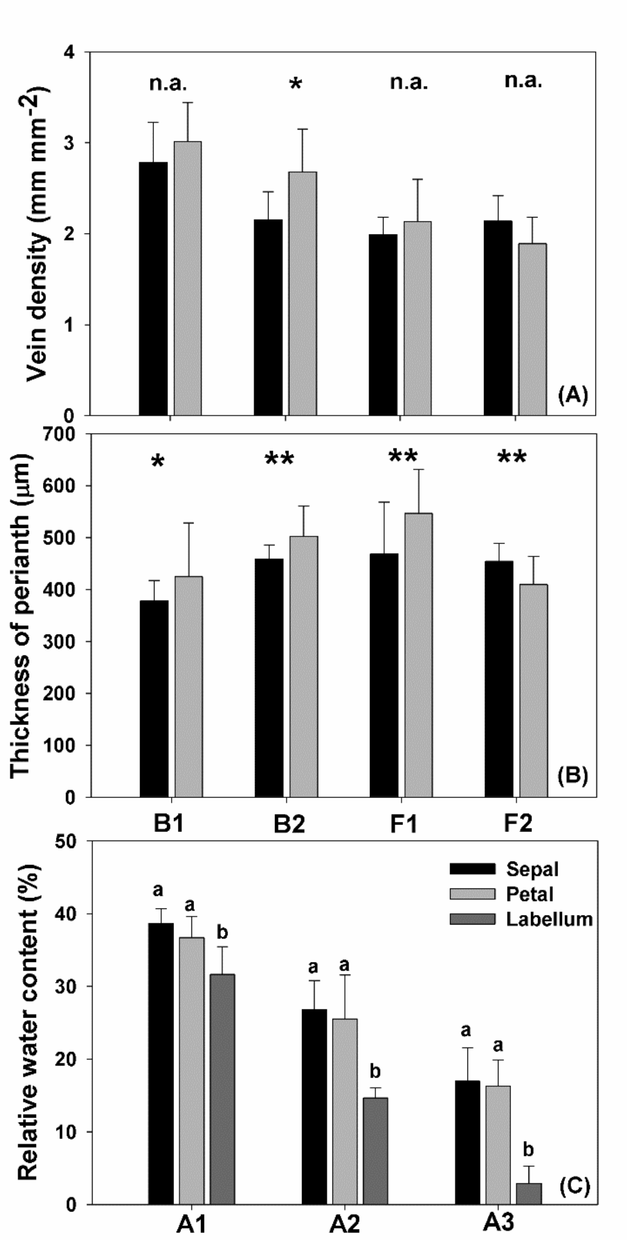

Figure S6 Anatomical differences in the flowers of *Dendrobium* 'Garnet Beauty' during development. Vein density (A), thickness of perianth (B), and relative water content (C). Statistical differences between sepals and petals at different stages of ageing were determined with independent-sample t tests. $*P < 0.05$; $**P < 0.01$, $***P < 0.001$. Different letters above bars indicate significant differences between sepals and petal at different stages of ageing. $P < 0.05$, based on ANOVA, followed by Tukey's post hoc tests for comparison. B1: budding stage 1, B2: budding stage 2, F1: flowering stage 1, F2: flowering stage 2, A1: ageing stage 1, A2: ageing stage 2, A3: ageing stage 3.

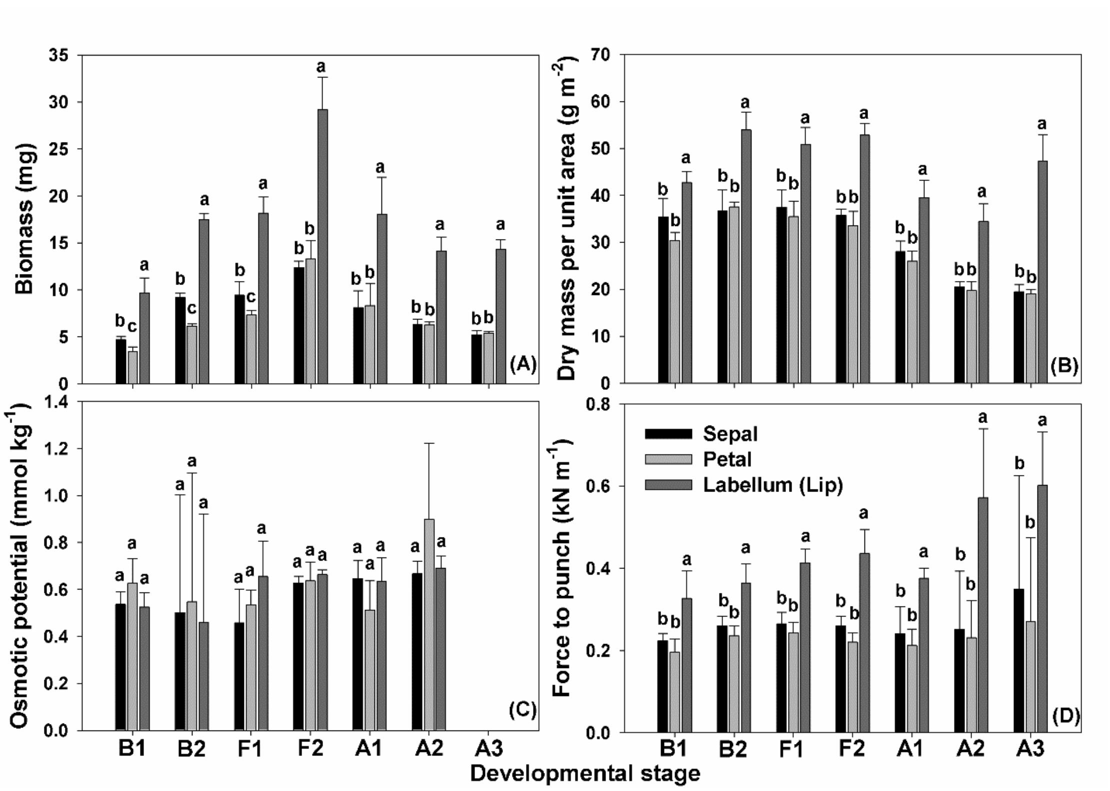

Figure S7 Anatomical and physiological differences between sepals, petals and labella in flower of *Dendrobium* 'Garnet Beauty' during development. Biomass of different perianth (A), dry mass per unit area (B), osmotic potential (C) and force to punch (D). Different letters above bars indicate significant differences between sepal, petal and labellum for different ageing processes. $P < 0.05$, based on ANOVA, followed by Tukey's post hoc tests for comparison. B1: budding stage 1, B2: budding stage 2, F1: flowering stage 1, F2: flowering stage 2, A1: ageing stage 1, A2: ageing stage 2, A3: ageing stage 3.

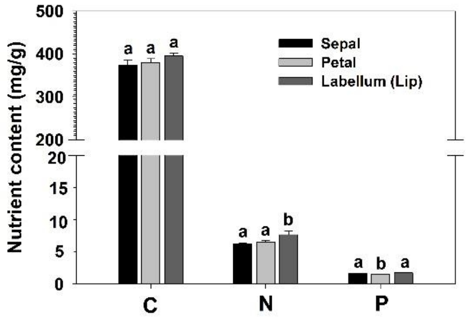

Figure S8 Nutrient contents of sepals, petals and labella. Different letters above bars indicate significant differences between sepals, petals and labella. $P < 0.05$, based on ANOVA, followed by Tukey's post hoc tests for comparison.

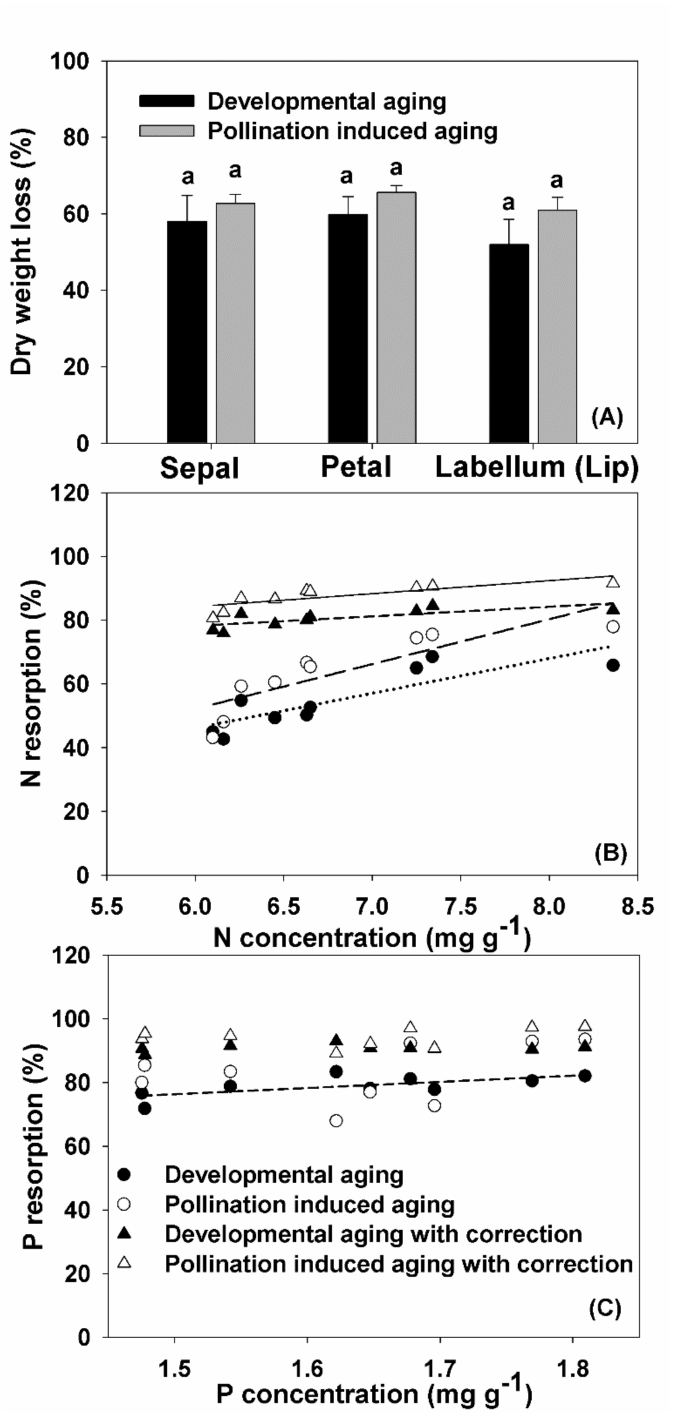

Figure S9 Dry weight loss of sepals, petals and labella during natural ageing and pollination-induced ageing (A), correlation of nitrogen concentration and N resorption efficiency as calculated when corrected and uncorrected for dry mass loss (B), correlation of phosphorus (P) concentration and P resorption efficiency as calculated when corrected and uncorrected for dry mass loss (C). Same letters above bars indicate no significant differences between sepals, petals and labella. $P < 0.05$, based on ANOVA, followed by Tukey's post hoc tests for comparison.

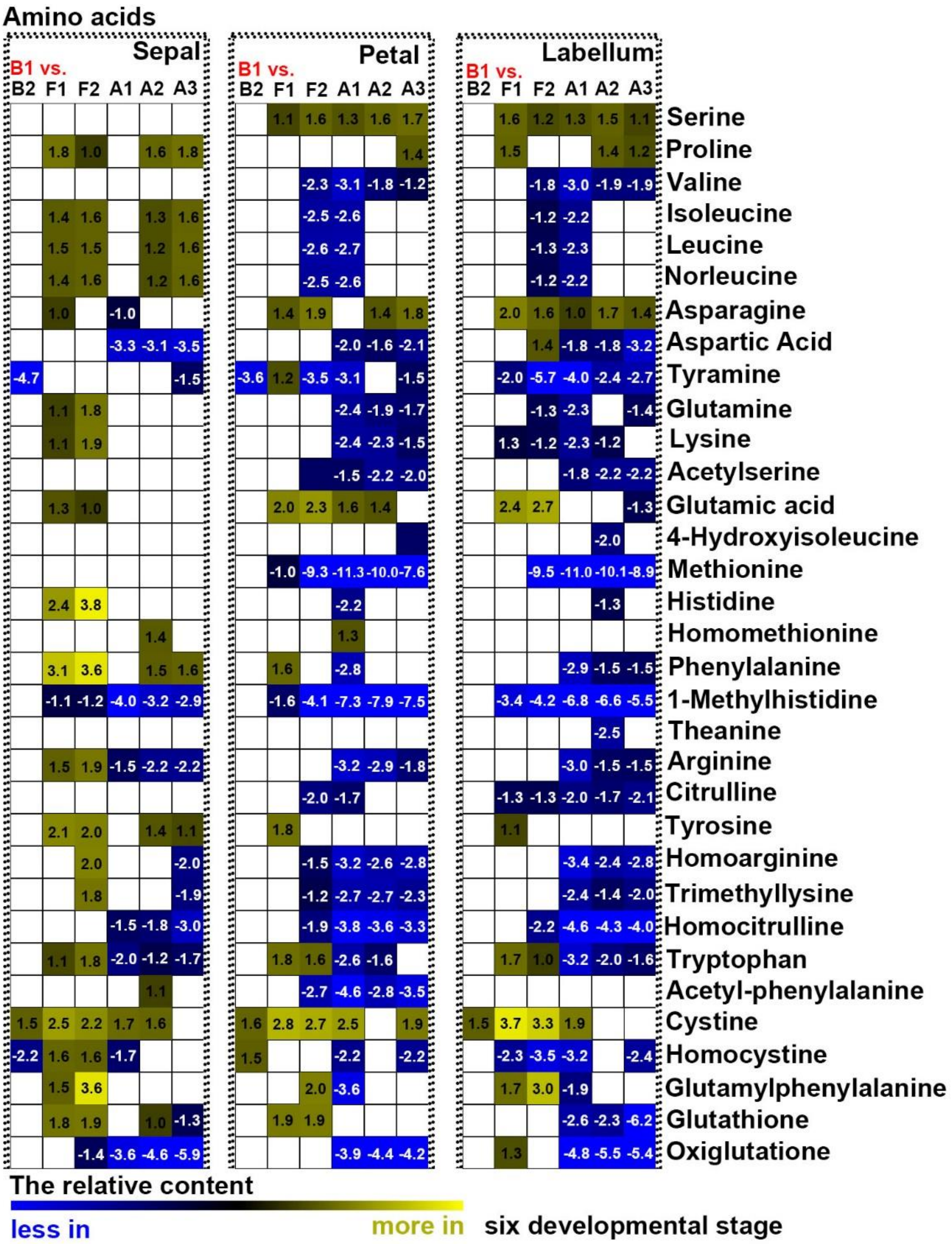

Figure S10 Abundance in amino acids detected by widely-targeted UPLC-MC for comparisons during flower development, all other stages were compared to budding stage 1 (B1). Numbers on heat map indicate the fold change between groups under comparison. White indicates no significant difference between groups under comparison. B2: budding stage 2, F1: flowering stage 1, F2: flowering stage 2, A1: ageing stage 1, A2: ageing stage 2, A3: ageing stage 3.

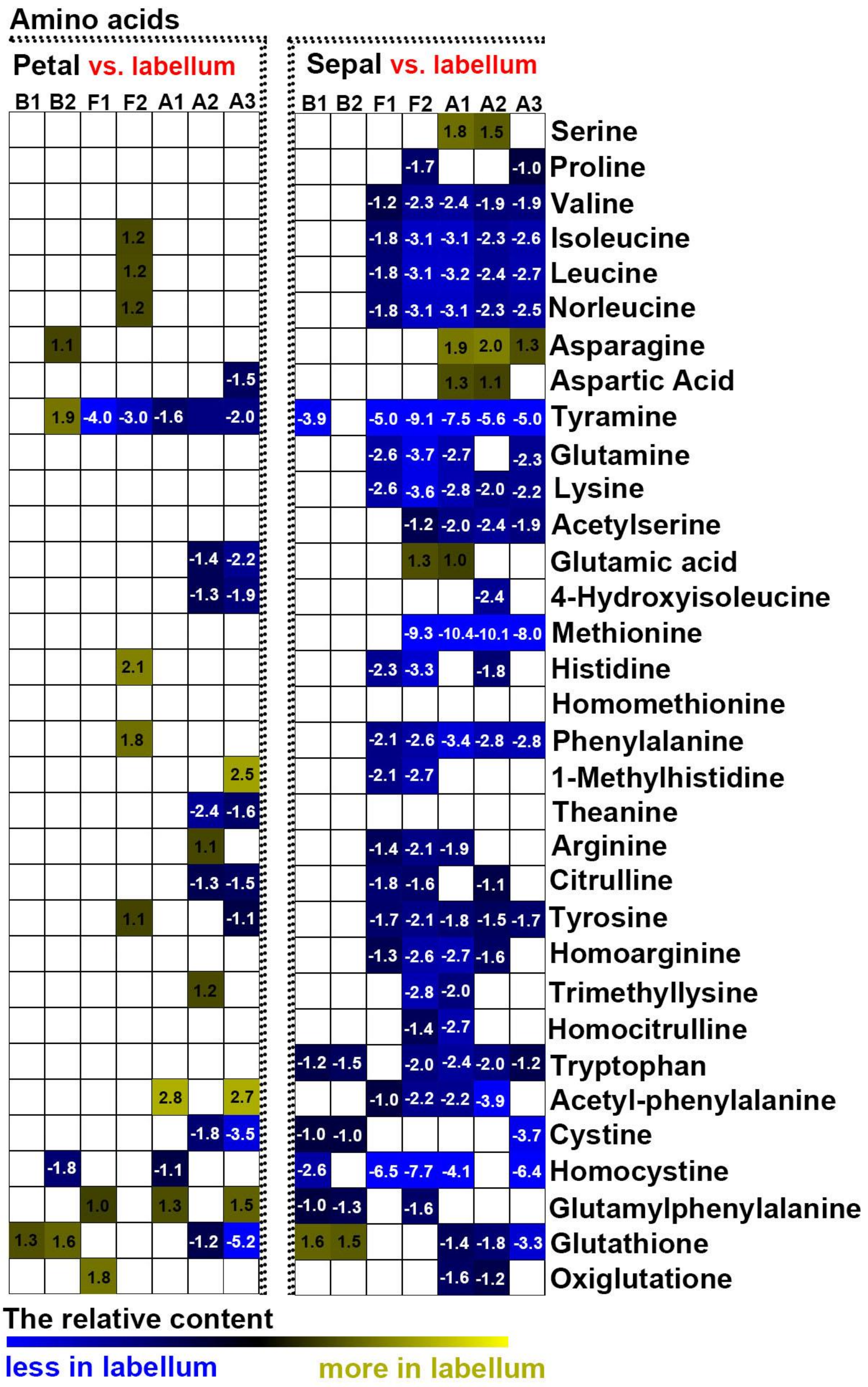

Figure S11 Abundance in amino acids detected by widely-targeted UPLC-MC for comparisons sepal to labellum or petal to labellum during flower development. Numbers on heat map indicate the fold change between groups under comparison. White indicates no significant difference between groups under comparison. B1: budding stage 1, B2: budding stage 2, F1: flowering stage 1, F2: flowering stage 2, A1: ageing stage 1, A2: ageing stage 2, A3: ageing stage 3.
